# Supplementary material for: Structure of tick-borne encephalitis virus and its neutralization by a monoclonal antibody
Source: Nat Commun. 2018 Jan 30;9:436. doi: 10.1038/s41467-018-02882-0 (PMC5789857; doi:10.1038/s41467-018-02882-0)
Supplement: Supplementary file 1 — Supplementary figures 1-10 [file 41467_2018_2882_MOESM1_ESM.pdf]

Supplementary information

Structure of tick-borne encephalitis virus and its neutralization by a monoclonal antibody

Füzik et al.

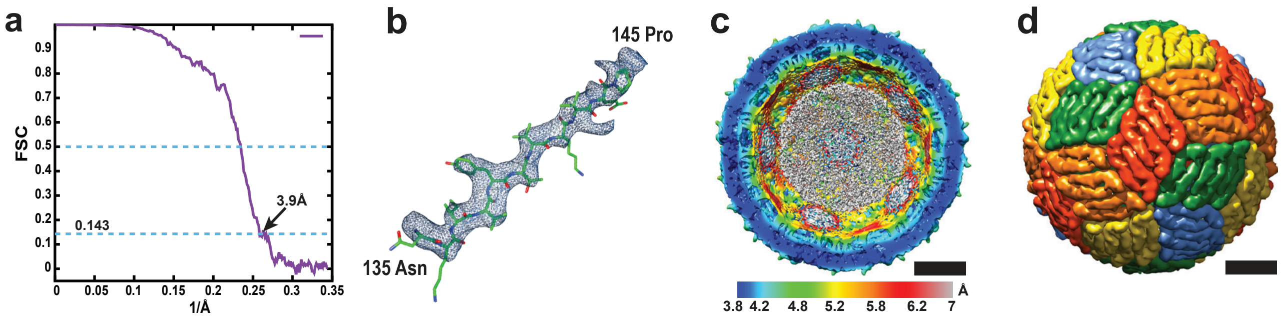

Supplementary Figure 1. Cryo-EM reconstruction of TBEV virion. (a) Fourier shell correlation curve of final reconstruction of TBEV virion calculated according to "gold standard". (b) Example electron density map of E-protein with corresponding structure. (c) Local resolution of cryo-EM map of TBEV virion. The display shows a cut-away half map colored according to the local resolution. The best resolved rigid parts include the ectodomains of the E-proteins. In contrast the virus membrane was reconstructed with less detail. Parts of the map with resolution worse than 7 Å are shown in grey. The non-sharpened electron density map was used for the display. (d) Molecular surface of TBEV virion low-pass filtered to 20 Å to show "Herringbone" organization of envelope proteins. Three dimers of E-proteins form a "raft" that is characteristic for flaviviruses. Scale bars in (c) and (d) represent 10 nm.

E-protein A - A2 interfaces

<table>
<tr><th colspan="4">Hydrogen bonds</th><th colspan="4">Salt bridges</th></tr>
<tr><th>##</th><th>Structure 1</th><th>Dist. [Å]</th><th>Structure 2</th><th>##</th><th>Structure 1</th><th>Dist. [Å]</th><th>Structure 2</th></tr>
<tr><td>1</td><td>A2:GLU 387[OE1]</td><td>3.57</td><td>A:LYS 309[NZ]</td><td>1</td><td>A2:GLU 387[OE1]</td><td>3.57</td><td>A:LYS 309[NZ]</td></tr>
</table>

Interface area: 420 Å^2

Interfacing residues

Inaccessible residues

Solvent-accessible residues

HSDC Residues making **H**ydrogen/**D**isulphide bond, **S**alt bridge or **C**ovalent link

Interfacing residues

ASA Accessible Surface Area, Å² **BSA** Buried Surface Area, Å² **$\Delta^i G$** Solvation energy effect, kcal/mol |||| Buried area percentage, one bar per 10%

<table>
<tr><th>##</th><th>Structure 1</th><th>HSDC</th><th>ASA</th><th>BSA</th><th>ΔiG</th></tr>
<tr><td>310</td><td>A2:THR 310</td><td></td><td>92.39</td><td>13.41 ||</td><td>0.03</td></tr>
<tr><td>311</td><td>A2:LYS 311</td><td></td><td>90.90</td><td>0.75 |</td><td>0.01</td></tr>
<tr><td>312</td><td>A2:PHE 312</td><td></td><td>6.25</td><td>4.41 |||||||</td><td>-0.05</td></tr>
<tr><td>313</td><td>A2:THR 313</td><td></td><td>81.35</td><td>65.64 ||||||||</td><td>0.99</td></tr>
<tr><td>314</td><td>A2:TRP 314</td><td></td><td>63.31</td><td>60.74 |||||||||</td><td>0.20</td></tr>
<tr><td>315</td><td>A2:LYS 315</td><td></td><td>134.73</td><td>12.48 |</td><td>0.05</td></tr>
<tr><td>317</td><td>A2:ALA 317</td><td></td><td>60.75</td><td>23.29 ||||</td><td>0.35</td></tr>
<tr><td>319</td><td>A2:THR 319</td><td></td><td>68.29</td><td>1.47 |</td><td>-0.02</td></tr>
<tr><td>333</td><td>A2:SER 333</td><td></td><td>86.20</td><td>1.73 |</td><td>-0.01</td></tr>
<tr><td>347</td><td>A2:HIS 347</td><td></td><td>106.13</td><td>59.61 ||||||</td><td>0.46</td></tr>
<tr><td>348</td><td>A2:GLY 348</td><td></td><td>62.96</td><td>12.87 |||</td><td>0.18</td></tr>
<tr><td>349</td><td>A2:SER 349</td><td></td><td>116.52</td><td>44.25 ||||</td><td>0.43</td></tr>
<tr><td>350</td><td>A2:PRO 350</td><td></td><td>40.31</td><td>9.02 |||</td><td>0.14</td></tr>
<tr><td>387</td><td>A2:GLU 387</td><td>HS</td><td>116.75</td><td>34.98 |||</td><td>0.03</td></tr>
<tr><td>388</td><td>A2:LEU 388</td><td></td><td>40.33</td><td>38.58 |||||||||</td><td>0.62</td></tr>
<tr><td>389</td><td>A2:SER 389</td><td></td><td>54.64</td><td>3.40 |</td><td>-0.04</td></tr>
<tr><td>390</td><td>A2:HIS 390</td><td></td><td>62.88</td><td>6.38 ||</td><td>-0.03</td></tr>
<tr><td>391</td><td>A2:GLN 391</td><td></td><td>121.61</td><td>24.42 |||</td><td>-0.27</td></tr>
<tr><td>395</td><td>A2:LYS 395</td><td></td><td>139.92</td><td>10.30 |</td><td>-0.08</td></tr>
</table>

<table>
<tr><th>##</th><th>Structure 2</th><th>HSDC</th><th>ASA</th><th>BSA</th><th>ΔiG</th></tr>
<tr><td>16</td><td>A:GLN 16</td><td></td><td>144.39</td><td>11.95 |</td><td>0.15</td></tr>
<tr><td>302</td><td>A:LEU 302</td><td></td><td>100.55</td><td>50.75 ||||||</td><td>0.43</td></tr>
<tr><td>303</td><td>A:THR 303</td><td></td><td>134.11</td><td>44.75 ||||</td><td>0.36</td></tr>
<tr><td>304</td><td>A:TYR 304</td><td></td><td>57.44</td><td>3.33 |</td><td>-0.02</td></tr>
<tr><td>305</td><td>A:THR 305</td><td></td><td>96.64</td><td>26.15 |||</td><td>0.42</td></tr>
<tr><td>306</td><td>A:MET 306</td><td></td><td>98.65</td><td>46.64 |||||</td><td>0.69</td></tr>
<tr><td>309</td><td>A:LYS 309</td><td>HS</td><td>114.39</td><td>30.84 |||</td><td>-1.05</td></tr>
<tr><td>339</td><td>A:ARG 339</td><td></td><td>67.64</td><td>50.60 ||||||||</td><td>-1.12</td></tr>
<tr><td>349</td><td>A:SER 349</td><td></td><td>116.52</td><td>10.80 |</td><td>-0.12</td></tr>
<tr><td>350</td><td>A:PRO 350</td><td></td><td>40.31</td><td>6.12 ||</td><td>-0.05</td></tr>
<tr><td>351</td><td>A:ASP 351</td><td></td><td>144.98</td><td>31.67 |||</td><td>0.05</td></tr>
<tr><td>352</td><td>A:VAL 352</td><td></td><td>93.60</td><td>56.65 |||||||</td><td>0.91</td></tr>
<tr><td>353</td><td>A:ASN 353</td><td></td><td>78.79</td><td>38.18 |||||</td><td>-0.07</td></tr>
</table>

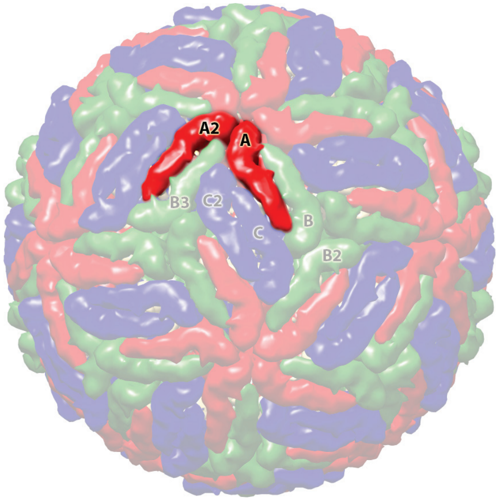

E-protein B - B2 interfaces

Hydrogen bonds

##	Structure 1	Dist. [Å]	Structure 2
1	B2:SER 168[N]	2.93	B:ASP 351[O]
2	B2:ASN 135[OD1]	2.07	B:ASP 351[N]
3	B2:GLU 295[OE2]	3.79	B:GLN 16[NE2]

Interface area: 410 Å^2

Interfacing residues

Inaccessible residues

Solvent-accessible residues

HSDC Residues making **H**ydrogen/**D**isulphide bond, **S**alt bridge or **C**ovalent link

Interfacing residues

ASA Accessible Surface Area, Å² **BSA** Buried Surface Area, Å² **$\Delta^i G$** Solvation energy effect, kcal/mol |||| Buried area percentage, one bar per 10%

<table>
<tr><th>##</th><th>Structure 1</th><th>HSDC</th><th>ASA</th><th>BSA</th><th>ΔiG</th></tr>
<tr><td>19</td><td>B2:THR 19</td><td></td><td>48.51</td><td>6.19 ||</td><td>0.10</td></tr>
<tr><td>20</td><td>B2:ARG 20</td><td></td><td>115.47</td><td>46.75 |||||</td><td>-0.04</td></tr>
<tr><td>134</td><td>B2:ALA 134</td><td></td><td>53.07</td><td>1.34 |</td><td>0.02</td></tr>
<tr><td>135</td><td>B2:ASN 135</td><td>H</td><td>105.24</td><td>58.71 ||||||</td><td>-0.54</td></tr>
<tr><td>167</td><td>B2:VAL 167</td><td></td><td>69.50</td><td>56.09 ||||||||</td><td>0.57</td></tr>
<tr><td>168</td><td>B2:SER 168</td><td>H</td><td>95.27</td><td>50.21 ||||||</td><td>0.65</td></tr>
<tr><td>170</td><td>B2:GLU 170</td><td></td><td>86.98</td><td>25.78 |||</td><td>-0.29</td></tr>
<tr><td>172</td><td>B2:THR 172</td><td></td><td>50.35</td><td>4.18 |</td><td>0.07</td></tr>
<tr><td>173</td><td>B2:ILE 173</td><td></td><td>107.75</td><td>55.78 ||||||</td><td>0.78</td></tr>
<tr><td>175</td><td>B2:THR 175</td><td></td><td>93.85</td><td>8.52 |</td><td>-0.09</td></tr>
<tr><td>181</td><td>B2:ASP 181</td><td></td><td>44.88</td><td>7.98 ||</td><td>-0.14</td></tr>
<tr><td>189</td><td>B2:ALA 189</td><td></td><td>95.69</td><td>11.55 ||</td><td>0.18</td></tr>
<tr><td>295</td><td>B2:GLU 295</td><td>H</td><td>95.83</td><td>11.78 ||</td><td>-0.12</td></tr>
<tr><td>296</td><td>B2:LYS 296</td><td></td><td>134.29</td><td>24.77 ||</td><td>-0.42</td></tr>
</table>

<table>
<tr><th>##</th><th>Structure 2</th><th>HSDC</th><th>ASA</th><th>BSA</th><th>ΔiG</th></tr>
<tr><td>16</td><td>B:GLN 16</td><td>H</td><td>154.11</td><td>87.11 ||||||</td><td>-0.15</td></tr>
<tr><td>37</td><td>B:GLY 37</td><td></td><td>50.45</td><td>4.51 |</td><td>-0.04</td></tr>
<tr><td>38</td><td>B:LYS 38</td><td></td><td>52.11</td><td>0.65 |</td><td>0.01</td></tr>
<tr><td>297</td><td>B:LEU 297</td><td></td><td>11.71</td><td>3.93 ||||</td><td>-0.04</td></tr>
<tr><td>298</td><td>B:LYS 298</td><td></td><td>124.19</td><td>9.20 |</td><td>0.15</td></tr>
<tr><td>302</td><td>B:LEU 302</td><td></td><td>103.21</td><td>47.31 |||||</td><td>0.57</td></tr>
<tr><td>303</td><td>B:THR 303</td><td></td><td>134.07</td><td>15.90 ||</td><td>0.25</td></tr>
<tr><td>339</td><td>B:ARG 339</td><td></td><td>69.62</td><td>14.99 |||</td><td>-0.50</td></tr>
<tr><td>343</td><td>B:ARG 343</td><td></td><td>102.14</td><td>33.67 ||||</td><td>0.21</td></tr>
<tr><td>345</td><td>B:VAL 345</td><td></td><td>8.21</td><td>0.67 |</td><td>0.01</td></tr>
<tr><td>350</td><td>B:PRO 350</td><td></td><td>84.30</td><td>22.18 |||</td><td>0.34</td></tr>
<tr><td>351</td><td>B:ASP 351</td><td>H</td><td>144.19</td><td>138.85 |||||||||</td><td>-0.59</td></tr>
<tr><td>352</td><td>B:VAL 352</td><td></td><td>76.68</td><td>29.46 ||||</td><td>0.47</td></tr>
<tr><td>353</td><td>B:ASN 353</td><td></td><td>76.29</td><td>37.36 |||||</td><td>0.14</td></tr>
</table>

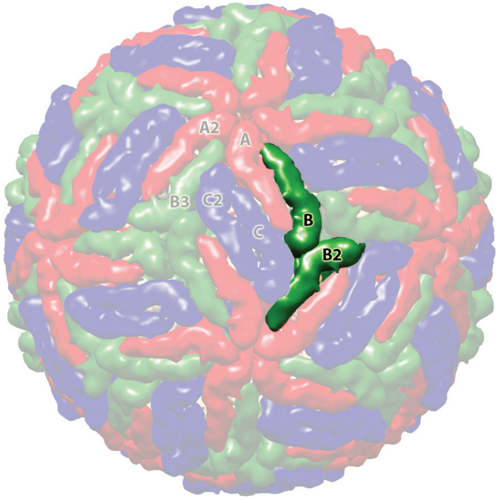

E-protein A - B3 interfaces

Interface area: 235 Å^2

Interfacing residues

Inaccessible residues

Solvent-accessible residues

HSDC Residues making **H**ydrogen/**D**isulphide bond, **S**alt bridge or **C**ovalent link

Interfacing residues

ASA Accessible Surface Area, Å² **BSA** Buried Surface Area, Å² **$\Delta^i G$** Solvation energy effect, kcal/mol |||| Buried area percentage, one bar per 10%

<table>
<tr><th>##</th><th>Structure 1</th><th>HSDC</th><th>ASA</th><th>BSA</th><th>ΔiG</th></tr>
<tr><td>73</td><td>B3:ARG 73</td><td></td><td>101.91</td><td>20.38 ||</td><td>-0.06</td></tr>
<tr><td>76</td><td>B3:THR 76</td><td></td><td>96.76</td><td>77.09 ||||||||</td><td>0.61</td></tr>
<tr><td>77</td><td>B3:MET 77</td><td></td><td>123.35</td><td>69.98 ||||||</td><td>1.50</td></tr>
<tr><td>78</td><td>B3:GLY 78</td><td></td><td>40.36</td><td>25.63 |||||||</td><td>0.23</td></tr>
<tr><td>79</td><td>B3:PRO 79</td><td></td><td>92.16</td><td>6.36 |</td><td>0.10</td></tr>
<tr><td>107</td><td>B3:LEU 107</td><td></td><td>107.17</td><td>38.50 ||||</td><td>0.62</td></tr>
</table>

<table>
<tr><th>##</th><th>Structure 2</th><th>HSDC</th><th>ASA</th><th>BSA</th><th>ΔiG</th></tr>
<tr><td>16</td><td>A:GLN 16</td><td></td><td>144.39</td><td>44.86 ||||</td><td>-0.39</td></tr>
<tr><td>17</td><td>A:GLY 17</td><td></td><td>67.94</td><td>20.37 |||</td><td>0.18</td></tr>
<tr><td>19</td><td>A:THR 19</td><td></td><td>44.66</td><td>27.64 |||||||</td><td>0.44</td></tr>
<tr><td>20</td><td>A:ARG 20</td><td></td><td>102.77</td><td>4.52 |</td><td>0.07</td></tr>
<tr><td>38</td><td>A:LYS 38</td><td></td><td>43.45</td><td>18.44 |||||</td><td>-0.43</td></tr>
<tr><td>295</td><td>A:GLU 295</td><td></td><td>82.96</td><td>70.35 ||||||||</td><td>0.33</td></tr>
<tr><td>296</td><td>A:LYS 296</td><td></td><td>127.50</td><td>43.50 ||||</td><td>-0.62</td></tr>
<tr><td>297</td><td>A:LEU 297</td><td></td><td>11.61</td><td>0.86 |</td><td>-0.01</td></tr>
</table>

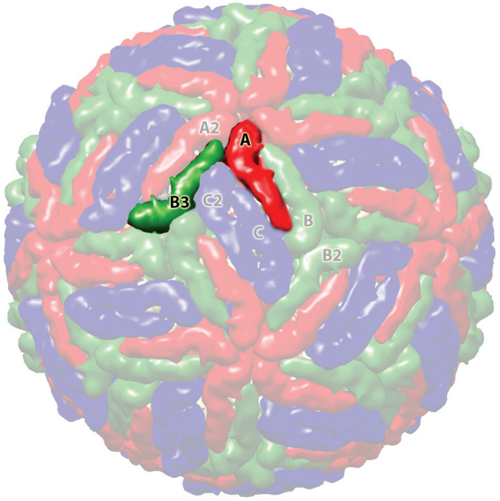

Supplementary Figure 2. Non-quasi equivalent E-protein interfaces in TBEV particle. The tables show hydrogen bonds, salt bridges and interfacing amino acids with buried surfaces participating in E-protein interactions. The interfaces are shown on the TBEV particles on the right side of the figure. Figure continued on next page.....

E-protein A - C2 interfaces

Hydrogen bonds

##	Structure 1	Dist. [Å]	Structure 2
1	C2:GLN 391[N]	2.51	A:VAL 167[O]
2	C2:GLN 391[NE2]	2.97	A:SER 169[O]
3	C2:ASP 380[OD2]	3.52	A:SER 190[OG]

Salt bridges

##	Structure 1	Dist. [Å]	Structure 2
1	C2:HIS 347[NE2]	3.21	A:GLU 291[OE1]
2	C2:HIS 347[ND1]	3.71	A:GLU 291[OE2]
3	C2:HIS 347[NE2]	2.64	A:GLU 291[OE2]
4	C2:ASP 380[OD2]	3.73	A:ARG 187[NE]

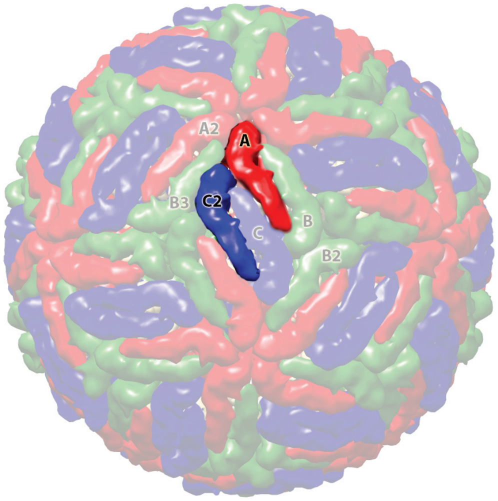

Interface area: 470 Å²

Interfacing residues

Inaccessible residues — HSDC Residues making **H**ydrogen/**D**isulphide bond, **S**alt bridge or **C**ovalent link

Solvent-accessible residues — Interfacing residues

ASA Accessible Surface Area, Å² **BSA** Buried Surface Area, Å² **ΔⁱG** Solvation energy effect, kcal/mol |||| Buried area percentage, one bar per 10%

##	Structure 1	HSDC	ASA	BSA	ΔⁱG
314	C2:TRP 314		65.08	5.58	0.01
317	C2:ALA 317		60.00	13.56	0.22
318	C2:PRO 318		8.71	8.22	-0.09
319	C2:THR 319		61.24	3.68	0.06
347	C2:HIS 347	S	149.57	80.08	0.60
380	C2:ASP 380	HS	84.95	52.29	-0.39
382	C2:ILE 382		26.60	16.55	0.26
384	C2:TYR 384		56.91	24.19	0.08
387	C2:GLU 387		116.52	11.10	-0.09
388	C2:LEU 388		40.07	1.34	0.02
389	C2:SER 389		55.80	47.80	-0.38
390	C2:HIS 390		59.88	52.88	-0.12
391	C2:GLN 391	H	129.68	121.96	0.15
392	C2:TRP 392		34.38	5.94	0.07
393	C2:PHE 393		127.41	29.08	0.42

##	Structure 2	HSDC	ASA	BSA	ΔⁱG
135	A:ASN 135		102.88	38.05	-0.25
166	A:THR 166		38.97	0.37	-0.00
167	A:VAL 167	H	83.73	70.48	0.58
168	A:SER 168		104.25	44.79	0.54
169	A:SER 169	H	36.75	11.32	-0.10
170	A:GLU 170		144.22	87.14	0.06
185	A:LEU 185		51.30	21.23	0.34
186	A:CYS 186		8.81	8.23	-0.09
187	A:ARG 187	S	104.97	90.72	-0.40
188	A:VAL 188		10.42	7.09	0.00
189	A:ALA 189		100.01	57.20	0.82
190	A:SER 190	H	42.16	17.50	0.27
291	A:GLU 291	S	28.77	9.82	-0.15

E-protein C - C2 interfaces

Hydrogen bonds

##	Structure 1	Dist. [Å]	Structure 2
1	C2:GLY 102[N]	3.18	C:TYR 150[OH]
2	C2:HIS 208[ND1]	3.70	C:VAL 254[O]
3	C2:HIS 208[NE2]	3.22	C:LEU 65[O]
4	C2:ASN 256[N]	2.63	C:HIS 208[O]
5	C2:LYS 266[NZ]	2.49	C:GLN 260[OE1]
6	C2:TYR 150[OH]	3.18	C:GLY 102[N]
7	C2:VAL 254[O]	3.70	C:HIS 208[ND1]
8	C2:LEU 65[O]	3.22	C:HIS 208[NE2]
9	C2:HIS 208[O]	2.63	C:ASN 256[N]
10	C2:GLN 260[OE1]	2.49	C:LYS 266[NZ]

Interface area: 1570 Å²

Interfacing residues

Inaccessible residues — HSDC Residues making **H**ydrogen/**D**isulphide bond, **S**alt bridge or **C**ovalent link

Solvent-accessible residues — Interfacing residues

ASA Accessible Surface Area, Å² **BSA** Buried Surface Area, Å² **ΔⁱG** Solvation energy effect, kcal/mol |||| Buried area percentage, one bar per 10%

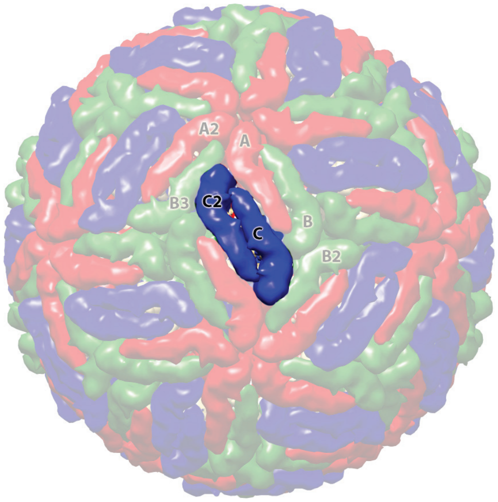

##	Structure 1	HSDC	ASA	BSA	ΔⁱG
4	C2:THR 4		29.46	22.95	-0.06
5	C2:HIS 5		64.51	23.19	0.02
7	C2:GLU 7		159.27	34.61	0.06
62	C2:HIS 62		51.27	8.13	0.13
65	C2:LEU 65	H	45.97	29.64	0.15
66	C2:SER 66		54.34	5.52	0.09
67	C2:ASP 67		85.01	0.14	-0.00
68	C2:THR 68		78.34	17.64	-0.20
98	C2:ASP 98		93.78	41.72	-0.24
100	C2:GLY 100		16.74	13.23	0.21
101	C2:TRP 101		167.93	132.06	1.51
102	C2:GLY 102	H	72.84	69.19	0.52
103	C2:ASN 103		29.57	2.14	-0.00
104	C2:HIS 104		161.67	37.00	-0.11
106	C2:GLY 106		54.47	5.40	-0.06
107	C2:LEU 107		104.44	21.41	0.34
108	C2:PHE 108		137.30	106.92	1.61
110	C2:LYS 110		101.89	1.96	-0.01
117	C2:VAL 117		2.26	0.84	0.01
125	C2:LYS 125		67.49	9.60	-0.36
150	C2:TYR 150	H	79.73	35.44	0.02
152	C2:ALA 152		46.09	34.81	0.56
153	C2:ALA 153		83.36	16.65	0.16
154	C2:ASN 154		134.90	19.33	-0.13
155	C2:GLU 155		104.11	1.56	0.02
206	C2:VAL 206		67.32	4.85	0.08
207	C2:GLU 207		145.95	2.21	-0.03
208	C2:HIS 208	H	163.42	144.51	0.39
209	C2:LEU 209		55.06	29.22	0.45
210	C2:PRO 210		59.48	33.09	0.53
225	C2:LEU 225		27.25	4.02	0.06
241	C2:LEU 241		2.01	0.84	0.01
254	C2:VAL 254	H	32.62	22.83	-0.15
255	C2:TYR 255		114.63	64.91	0.84
256	C2:ASN 256	H	59.13	46.64	-0.05
257	C2:LEU 257		83.65	49.54	0.48
258	C2:GLY 258		24.08	18.73	0.30
259	C2:ASP 259		60.98	56.80	-0.06
260	C2:GLN 260	H	33.63	28.56	-0.33
261	C2:THR 261		26.31	12.38	0.20
262	C2:GLY 262		44.24	43.07	0.48
263	C2:VAL 263		97.65	47.52	0.76
265	C2:LEU 265		59.16	36.75	0.56
266	C2:LYS 266	H	173.11	71.14	-0.53
273	C2:VAL 273		68.14	4.35	0.07
316	C2:ARG 316		81.38	66.63	-2.36
317	C2:ALA 317		60.00	0.12	-0.00
319	C2:THR 319		61.24	43.72	0.17
320	C2:ASP 320		48.78	18.40	-0.11
321	C2:SER 321		26.59	22.22	0.13
322	C2:GLY 322		66.83	6.81	0.04
327	C2:VAL 327		6.02	4.60	0.07
329	C2:GLU 329		37.68	1.35	-0.02

##	Structure 2	HSDC	ASA	BSA	ΔⁱG
4	C:THR 4		29.46	23.44	-0.07
5	C:HIS 5		64.51	23.46	0.03
7	C:GLU 7		159.27	34.45	0.05
62	C:HIS 62		51.27	8.44	0.14
65	C:LEU 65	H	45.97	30.30	0.14
66	C:SER 66		54.34	5.69	0.09
67	C:ASP 67		85.01	0.29	-0.00
68	C:THR 68		78.34	17.39	-0.20
98	C:ASP 98		93.78	42.19	-0.25
100	C:GLY 100		16.74	13.39	0.21
101	C:TRP 101		167.93	131.12	1.51
102	C:GLY 102	H	72.84	68.41	0.51
103	C:ASN 103		29.57	1.84	-0.00
104	C:HIS 104		161.67	37.10	-0.11
106	C:GLY 106		54.47	5.60	-0.06
107	C:LEU 107		104.44	22.41	0.36
108	C:PHE 108		137.30	106.68	1.60
110	C:LYS 110		101.89	1.89	-0.03
117	C:VAL 117		2.26	0.67	0.01
125	C:LYS 125		67.49	9.08	-0.34
150	C:TYR 150	H	79.73	36.28	0.03
152	C:ALA 152		46.09	34.51	0.55
153	C:ALA 153		83.36	17.30	0.17
154	C:ASN 154		134.90	19.83	-0.14
155	C:GLU 155		104.11	1.24	0.02
206	C:VAL 206		67.32	4.69	0.08
207	C:GLU 207		145.95	2.95	-0.03
208	C:HIS 208	H	163.42	143.38	0.35
209	C:LEU 209		55.06	28.26	0.44
210	C:PRO 210		59.48	32.56	0.51
225	C:LEU 225		27.25	3.43	0.05
241	C:LEU 241		2.01	1.00	0.02
254	C:VAL 254	H	32.62	23.20	-0.15
255	C:TYR 255		114.63	65.62	0.84
256	C:ASN 256	H	59.13	45.83	-0.05
257	C:LEU 257		83.65	50.87	0.51
258	C:GLY 258		24.08	18.72	0.30
259	C:ASP 259		60.98	56.18	-0.07
260	C:GLN 260	H	33.63	23.07	-0.26
261	C:THR 261		26.31	12.04	0.19
262	C:GLY 262		44.24	43.50	0.47
263	C:VAL 263		97.65	49.05	0.78
265	C:LEU 265		59.16	36.28	0.55
266	C:LYS 266	H	173.11	71.43	-0.53
273	C:VAL 273		68.14	5.19	0.08
316	C:ARG 316		81.38	67.16	-2.34
319	C:THR 319		61.24	44.58	0.19
320	C:ASP 320		48.78	18.15	-0.12
321	C:SER 321		26.59	21.54	0.13
322	C:GLY 322		66.83	7.46	0.05
327	C:VAL 327		6.02	5.18	0.08
329	C:GLU 329		37.68	1.47	-0.02

Supplementary Figure 2 (continued). Non-quasi equivalent E-protein interfaces in TBEV particle. The tables show hydrogen bonds, salt bridges and interfacing amino acids with buried surfaces participating in E-protein interactions. The interfaces are shown on the TBEV particles on the right side of the figure. Figure continued on next page.....

E-protein A - C interfaces

Interface area: 330 Å²

Interfacing residues

Inaccessible residues
Solvent-accessible residues
HSDC Residues making **H**ydrogen/**D**isulphide bond, **S**alt bridge or **C**ovalent link
Interfacing residues
ASA Accessible Surface Area, Å² **BSA** Buried Surface Area, Å² **ΔⁱG** Solvation energy effect, kcal/mol |||| Buried area percentage, one bar per 10%

##	Structure 1	HSDC	ASA	BSA	ΔⁱG
54	C:ALA 54		74.08	7.87 ||	0.13
76	C:THR 76		101.27	8.42 |	-0.06
78	C:GLY 78		41.94	14.11 ||||	0.23
79	C:PRO 79		90.36	35.23 ||||	0.42
81	C:THR 81		90.49	14.92 ||	0.20
86	C:HIS 86		167.33	93.21 ||||||	-0.02
87	C:GLN 87		80.08	4.28 |	-0.04
88	C:GLY 88		43.41	13.21 ||||	0.18
89	C:GLY 89		15.74	4.95 ||||	-0.06
107	C:LEU 107		104.44	11.64 ||	0.19
135	C:ASN 135		101.97	10.43 ||	-0.12
229	C:HIS 229		84.52	54.11 |||||||	0.13
230	C:GLU 230		128.43	2.33 |	-0.03
232	C:ALA 232		38.78	14.56 ||||	0.23
234	C:ASN 234		66.25	17.93 |||	0.04
235	C:TRP 235		25.12	3.56 ||	-0.04
236	C:ASN 236		70.79	19.77 |||	-0.25

##	Structure 2	HSDC	ASA	BSA	ΔⁱG
54	A:ALA 54		73.75	8.22 ||	0.13
73	A:ARG 73		100.87	2.48 |	-0.03
76	A:THR 76		99.68	7.57 |	-0.08
78	A:GLY 78		42.78	19.26 |||||	0.31
79	A:PRO 79		88.06	31.12 ||||	0.32
81	A:THR 81		88.55	23.46 |||	0.26
86	A:HIS 86		165.17	98.54 ||||||	0.96
87	A:GLN 87		82.14	6.44 |	0.02
88	A:GLY 88		44.17	15.86 ||||	0.15
89	A:GLY 89		14.49	3.93 |||	-0.04
107	A:LEU 107		106.14	9.63 |	0.15
135	A:ASN 135		102.88	12.00 ||	-0.14
229	A:HIS 229		88.31	52.32 ||||||	0.13
232	A:ALA 232		39.90	14.24 ||||	0.23
234	A:ASN 234		66.57	17.93 |||	0.02
235	A:TRP 235		24.33	2.09 |	-0.02
236	A:ASN 236		71.56	7.05 |	-0.08

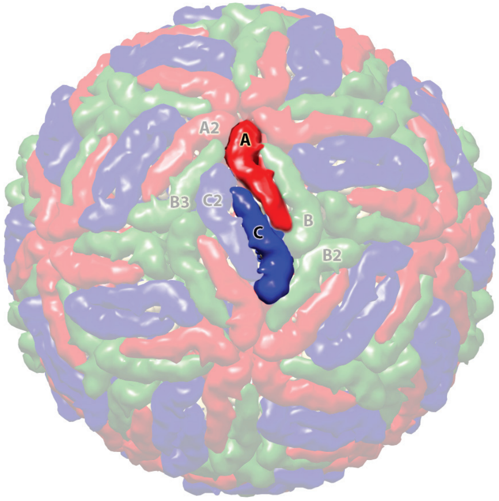

E-protein B - C interfaces

Hydrogen bonds

##	Structure 1	Dist. [Å]	Structure 2
1	C:VAL 167[O]	2.72	B:GLN 391[N]
2	C:SER 169[O]	3.43	B:GLN 391[NE2]
3	C:GLU 170[OE1]	3.54	B:TYR 384[OH]
4	C:GLU 291[OE1]	3.10	B:HIS 347[NE2]

Salt bridges

##	Structure 1	Dist. [Å]	Structure 2
1	C:ARG 187[NE]	3.85	B:ASP 380[OD2]
2	C:GLU 291[OE1]	3.10	B:HIS 347[NE2]
3	C:GLU 291[OE2]	3.93	B:HIS 347[NE2]

Interface area: 470 Å²

Interfacing residues

Inaccessible residues
Solvent-accessible residues
HSDC Residues making **H**ydrogen/**D**isulphide bond, **S**alt bridge or **C**ovalent link
Interfacing residues
ASA Accessible Surface Area, Å² **BSA** Buried Surface Area, Å² **ΔⁱG** Solvation energy effect, kcal/mol |||| Buried area percentage, one bar per 10%

##	Structure 1	HSDC	ASA	BSA	ΔⁱG
20	C:ARG 20		128.90	8.29 |	-0.09
135	C:ASN 135		101.97	40.61 ||||	-0.32
166	C:THR 166		41.55	0.37 |	-0.00
167	C:VAL 167	H	77.33	62.52 ||||||||	0.40
168	C:SER 168		102.99	43.16 |||||	0.51
169	C:SER 169	H	38.86	14.51 ||||	-0.11
170	C:GLU 170	H	140.63	79.18 ||||||	0.25
185	C:LEU 185		58.73	30.65 ||||||	0.49
186	C:CYS 186		7.64	6.26 |||||||||	-0.07
187	C:ARG 187	S	100.69	80.71 |||||||||	-0.59
188	C:VAL 188		11.27	7.10 |||||||	0.01
189	C:ALA 189		102.23	57.29 ||||||	0.82
190	C:SER 190		40.95	19.49 |||||	0.31
291	C:GLU 291	HS	31.26	14.15 |||||	-0.17

##	Structure 2	HSDC	ASA	BSA	ΔⁱG
314	B:TRP 314		71.47	5.50 |	-0.02
317	B:ALA 317		65.45	17.74 |||	0.28
318	B:PRO 318		8.80	6.62 ||||||||	-0.08
319	B:THR 319		68.89	2.01 |	0.03
347	B:HIS 347	HS	151.61	72.58 |||||	0.65
380	B:ASP 380	S	92.85	53.37 ||||||	-0.20
382	B:ILE 382		31.09	23.34 ||||||||	0.37
384	B:TYR 384	H	56.94	28.39 |||||	0.04
387	B:GLU 387		117.56	9.08 |	-0.05
388	B:LEU 388		41.44	1.84 |	0.03
389	B:SER 389		55.86	48.70 |||||||||	-0.37
390	B:HIS 390		62.45	54.10 |||||||||	-0.12
391	B:GLN 391	H	126.80	123.60 ||||||||||	0.10
392	B:TRP 392		36.44	6.23 ||	0.07
393	B:PHE 393		126.21	25.63 |||	0.37

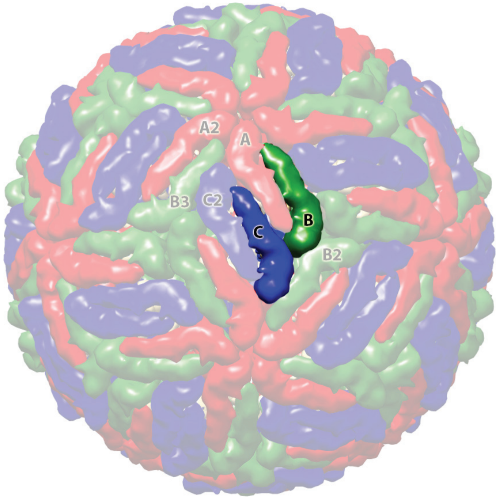

E-protein C - B2 interfaces

Salt bridges

##	Structure 1	Dist. [Å]	Structure 2
1	C:ASP 351[OD2]	3.18	B2:ARG 94[NE]
2	C:ASP 351[OD2]	3.15	B2:ARG 94[NH1]
3	C:ASP 351[OD2]	3.91	B2:ARG 94[NH2]

Interface area: 580 Å²

Interfacing residues

Inaccessible residues
Solvent-accessible residues
HSDC Residues making **H**ydrogen/**D**isulphide bond, **S**alt bridge or **C**ovalent link
Interfacing residues
ASA Accessible Surface Area, Å² **BSA** Buried Surface Area, Å² **ΔⁱG** Solvation energy effect, kcal/mol |||| Buried area percentage, one bar per 10%

##	Structure 1	HSDC	ASA	BSA	ΔⁱG
16	C:GLN 16		144.34	111.23 ||||||||	-0.00
17	C:GLY 17		63.69	2.51 |	0.04
36	C:GLU 36		107.96	3.44 |	-0.04
37	C:GLY 37		43.21	19.99 |||||	0.04
38	C:LYS 38		37.75	10.82 |||	-0.20
171	C:LYS 171		100.58	2.37 |	-0.09
295	C:GLU 295		92.73	23.57 |||	0.08
296	C:LYS 296		125.93	1.34 |	0.02
297	C:LEU 297		11.88	4.54 ||||	-0.05
298	C:LYS 298		122.60	18.41 ||	0.29
299	C:MET 299		42.54	2.87 |	0.01
302	C:LEU 302		103.28	78.11 ||||||||	0.88
303	C:THR 303		133.59	33.11 |||	0.37
304	C:TYR 304		60.40	0.61 |	-0.01
306	C:MET 306		104.04	34.57 ||||	0.68
309	C:LYS 309		117.23	42.04 ||||	-0.39
339	C:ARG 339		71.88	31.03 |||||	-0.75
343	C:ARG 343		102.64	41.94 |||||	0.34
345	C:VAL 345		8.18	1.67 |||	0.03
350	C:PRO 350		98.86	7.97 |	0.01
351	C:ASP 351	S	142.20	101.19 ||||||||	-0.57
353	C:ASN 353		78.52	14.73 ||	0.11

##	Structure 2	HSDC	ASA	BSA	ΔⁱG
54	B2:ALA 54		75.40	13.90 ||	0.22
56	B2:THR 56		29.41	0.34 |	0.01
57	B2:ARG 57		57.79	3.16 |	-0.01
81	B2:THR 81		91.51	17.91 ||	0.29
83	B2:ALA 83		73.87	28.37 ||||	0.41
85	B2:GLU 85		44.55	17.04 ||||	-0.16
86	B2:HIS 86		167.84	111.63 |||||||	0.33
87	B2:GLN 87		81.70	38.89 |||||	0.14
88	B2:GLY 88		42.00	9.30 |||	0.01
94	B2:ARG 94	S	113.45	25.86 |||	0.18
118	B2:LYS 118		81.47	0.26 |	-0.01
136	B2:LYS 136		133.30	4.04 |	-0.06
227	B2:TRP 227		54.30	11.88 |||	0.19
228	B2:LYS 228		19.10	0.50 |	0.01
229	B2:HIS 229		87.17	79.37 ||||||||||	1.10
230	B2:GLU 230		126.73	27.82 |||	-0.18
231	B2:GLY 231		81.35	45.94 ||||||	0.04
232	B2:ALA 232		38.31	16.67 |||||	0.25
233	B2:ARG 233		218.63	105.43 |||||	-0.32
234	B2:ASN 234		65.04	14.67 |||	-0.17
236	B2:ASN 236		71.34	0.12 |	-0.00

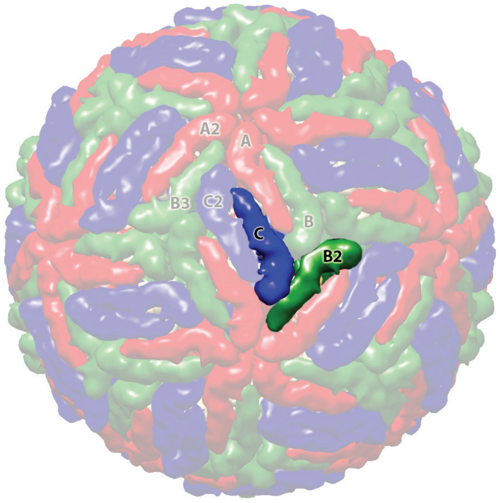

Supplementary Figure 2 (continued). Non-quasi equivalent E-protein interfaces in TBEV particle. The tables show hydrogen bonds, salt bridges and interfacing amino acids with buried surfaces participating in E-protein interactions. The interfaces are shown on the TBEV particles on the right side of the figure.

a

Hydrogen bonds

##	Structure 1	Dist. [Å]	Structure 2
1	B:HIS 208[NE2]	3.50	A:LEU 65[O]
2	B:GLY 102[N]	3.78	A:TYR 150[OH]
3	B:ASN 256[N]	2.86	A:HIS 208[O]
4	B:HIS 208[ND1]	3.70	A:VAL 254[O]
5	B:LYS 266[NZ]	2.68	A:GLN 260[OE1]
6	B:LEU 65[O]	3.38	A:HIS 208[NE2]
7	B:TYR 150[OH]	3.71	A:GLY 102[N]
8	B:HIS 208[O]	2.83	A:ASN 256[N]
9	B:VAL 254[O]	3.35	A:HIS 208[ND1]
10	B:GLN 260[OE1]	2.46	A:LYS 266[NZ]

Interface area: 1490 Å²

Interfacing residues

Inaccessible residues
Solvent-accessible residues
HSDC Residues making **H**ydrogen/**D**isulphide bond, **S**alt bridge or **C**ovalent link
Interfacing residues
ASA Accessible Surface Area, Å² **BSA** Buried Surface Area, Å² **ΔⁱG** Solvation energy effect, kcal/mol |||| Buried area percentage, one bar per 10%

<table>
<tr><th>##</th><th>Structure 1</th><th>HSDC</th><th>ASA</th><th>BSA</th><th>ΔⁱG</th></tr>
<tr><td>4</td><td>B:THR 4</td><td></td><td>33.35</td><td>25.41 ||||||||</td><td>-0.09</td></tr>
<tr><td>5</td><td>B:HIS 5</td><td></td><td>66.70</td><td>24.58 ||||</td><td>0.03</td></tr>
<tr><td>6</td><td>B:LEU 6</td><td></td><td>60.50</td><td>0.33 |</td><td>0.01</td></tr>
<tr><td>7</td><td>B:GLU 7</td><td></td><td>158.60</td><td>30.78 ||</td><td>0.18</td></tr>
<tr><td>62</td><td>B:HIS 62</td><td></td><td>50.40</td><td>5.32 ||</td><td>0.09</td></tr>
<tr><td>65</td><td>B:LEU 65</td><td>H</td><td>45.68</td><td>31.19 |||||||</td><td>0.13</td></tr>
<tr><td>66</td><td>B:SER 66</td><td></td><td>48.43</td><td>3.18 |</td><td>0.05</td></tr>
<tr><td>68</td><td>B:THR 68</td><td></td><td>77.63</td><td>16.98 |||</td><td>-0.19</td></tr>
<tr><td>98</td><td>B:ASP 98</td><td></td><td>95.69</td><td>38.21 ||||</td><td>-0.17</td></tr>
<tr><td>100</td><td>B:GLY 100</td><td></td><td>17.24</td><td>14.40 |||||||||</td><td>0.23</td></tr>
<tr><td>101</td><td>B:TRP 101</td><td></td><td>164.53</td><td>116.73 ||||||||</td><td>1.21</td></tr>
<tr><td>102</td><td>B:GLY 102</td><td>H</td><td>72.67</td><td>65.96 ||||||||||</td><td>0.49</td></tr>
<tr><td>103</td><td>B:ASN 103</td><td></td><td>30.66</td><td>0.15 |</td><td>-0.00</td></tr>
<tr><td>104</td><td>B:HIS 104</td><td></td><td>161.33</td><td>36.86 |||</td><td>-0.32</td></tr>
<tr><td>106</td><td>B:GLY 106</td><td></td><td>50.90</td><td>4.17 |</td><td>-0.05</td></tr>
<tr><td>107</td><td>B:LEU 107</td><td></td><td>107.17</td><td>23.09 |||</td><td>0.37</td></tr>
<tr><td>108</td><td>B:PHE 108</td><td></td><td>132.68</td><td>101.70 ||||||||</td><td>1.53</td></tr>
<tr><td>109</td><td>B:GLY 109</td><td></td><td>39.94</td><td>0.29 |</td><td>-0.00</td></tr>
<tr><td>110</td><td>B:LYS 110</td><td></td><td>103.14</td><td>0.79 |</td><td>-0.03</td></tr>
<tr><td>117</td><td>B:VAL 117</td><td></td><td>2.27</td><td>0.67 |||</td><td>0.01</td></tr>
<tr><td>125</td><td>B:LYS 125</td><td></td><td>66.92</td><td>5.40 |</td><td>-0.20</td></tr>
<tr><td>150</td><td>B:TYR 150</td><td>H</td><td>75.35</td><td>30.46 |||||</td><td>-0.00</td></tr>
<tr><td>152</td><td>B:ALA 152</td><td></td><td>41.77</td><td>28.96 |||||||</td><td>0.46</td></tr>
<tr><td>153</td><td>B:ALA 153</td><td></td><td>78.25</td><td>14.21 ||</td><td>0.14</td></tr>
<tr><td>154</td><td>B:ASN 154</td><td></td><td>130.58</td><td>26.79 |||</td><td>-0.20</td></tr>
<tr><td>206</td><td>B:VAL 206</td><td></td><td>68.39</td><td>2.34 |</td><td>0.04</td></tr>
<tr><td>207</td><td>B:GLU 207</td><td></td><td>144.25</td><td>1.47 |</td><td>-0.02</td></tr>
<tr><td>208</td><td>B:HIS 208</td><td>H</td><td>163.73</td><td>134.22 |||||||||</td><td>0.35</td></tr>
<tr><td>209</td><td>B:LEU 209</td><td></td><td>59.34</td><td>31.15 ||||||</td><td>0.47</td></tr>
<tr><td>210</td><td>B:PRO 210</td><td></td><td>53.32</td><td>26.51 |||||</td><td>0.41</td></tr>
<tr><td>225</td><td>B:LEU 225</td><td></td><td>25.60</td><td>0.50 |</td><td>0.01</td></tr>
<tr><td>254</td><td>B:VAL 254</td><td>H</td><td>31.47</td><td>22.54 ||||||||</td><td>-0.13</td></tr>
<tr><td>255</td><td>B:TYR 255</td><td></td><td>111.92</td><td>60.22 ||||||</td><td>0.81</td></tr>
<tr><td>256</td><td>B:ASN 256</td><td>H</td><td>59.66</td><td>43.28 ||||||||</td><td>-0.06</td></tr>
<tr><td>257</td><td>B:LEU 257</td><td></td><td>85.28</td><td>46.07 ||||||</td><td>0.45</td></tr>
<tr><td>258</td><td>B:GLY 258</td><td></td><td>25.59</td><td>17.89 |||||||</td><td>0.28</td></tr>
<tr><td>259</td><td>B:ASP 259</td><td></td><td>61.62</td><td>55.28 |||||||||</td><td>-0.05</td></tr>
<tr><td>260</td><td>B:GLN 260</td><td>H</td><td>33.66</td><td>22.65 |||||||</td><td>-0.26</td></tr>
<tr><td>261</td><td>B:THR 261</td><td></td><td>31.15</td><td>15.34 |||||</td><td>0.23</td></tr>
<tr><td>262</td><td>B:GLY 262</td><td></td><td>42.35</td><td>41.63 ||||||||||</td><td>0.45</td></tr>
<tr><td>263</td><td>B:VAL 263</td><td></td><td>95.11</td><td>51.06 ||||||</td><td>0.81</td></tr>
<tr><td>265</td><td>B:LEU 265</td><td></td><td>57.48</td><td>31.93 ||||||</td><td>0.47</td></tr>
<tr><td>266</td><td>B:LYS 266</td><td>H</td><td>173.13</td><td>67.13 ||||</td><td>-0.49</td></tr>
<tr><td>273</td><td>B:VAL 273</td><td></td><td>66.78</td><td>5.19 |</td><td>0.08</td></tr>
<tr><td>316</td><td>B:ARG 316</td><td></td><td>73.43</td><td>55.15 ||||||||</td><td>-0.01</td></tr>
<tr><td>317</td><td>B:ALA 317</td><td></td><td>65.45</td><td>1.03 |</td><td>-0.01</td></tr>
<tr><td>319</td><td>B:THR 319</td><td></td><td>68.89</td><td>52.90 ||||||||</td><td>0.33</td></tr>
<tr><td>320</td><td>B:ASP 320</td><td></td><td>48.31</td><td>16.72 ||||</td><td>-0.12</td></tr>
<tr><td>321</td><td>B:SER 321</td><td></td><td>28.01</td><td>24.36 |||||||||</td><td>0.09</td></tr>
<tr><td>322</td><td>B:GLY 322</td><td></td><td>67.50</td><td>5.23 |</td><td>0.03</td></tr>
<tr><td>327</td><td>B:VAL 327</td><td></td><td>10.87</td><td>10.20 ||||||||||</td><td>0.16</td></tr>
</table>

<table>
<tr><th>##</th><th>Structure 2</th><th>HSDC</th><th>ASA</th><th>BSA</th><th>ΔⁱG</th></tr>
<tr><td>4</td><td>A:THR 4</td><td></td><td>30.36</td><td>24.12 ||||||||</td><td>-0.06</td></tr>
<tr><td>5</td><td>A:HIS 5</td><td></td><td>65.89</td><td>25.98 ||||</td><td>0.01</td></tr>
<tr><td>7</td><td>A:GLU 7</td><td></td><td>156.70</td><td>30.83 ||</td><td>0.17</td></tr>
<tr><td>62</td><td>A:HIS 62</td><td></td><td>49.49</td><td>5.63 ||</td><td>0.09</td></tr>
<tr><td>65</td><td>A:LEU 65</td><td>H</td><td>47.79</td><td>30.50 |||||||</td><td>0.17</td></tr>
<tr><td>66</td><td>A:SER 66</td><td></td><td>57.60</td><td>5.35 |</td><td>0.09</td></tr>
<tr><td>68</td><td>A:THR 68</td><td></td><td>77.08</td><td>16.88 |||</td><td>-0.19</td></tr>
<tr><td>98</td><td>A:ASP 98</td><td></td><td>94.15</td><td>40.58 |||||</td><td>-0.23</td></tr>
<tr><td>100</td><td>A:GLY 100</td><td></td><td>17.58</td><td>13.56 ||||||||</td><td>0.22</td></tr>
<tr><td>101</td><td>A:TRP 101</td><td></td><td>164.50</td><td>119.04 ||||||||</td><td>1.24</td></tr>
<tr><td>102</td><td>A:GLY 102</td><td>H</td><td>72.29</td><td>65.39 ||||||||||</td><td>0.49</td></tr>
<tr><td>103</td><td>A:ASN 103</td><td></td><td>30.22</td><td>0.44 |</td><td>-0.00</td></tr>
<tr><td>104</td><td>A:HIS 104</td><td></td><td>158.62</td><td>34.92 |||</td><td>-0.17</td></tr>
<tr><td>106</td><td>A:GLY 106</td><td></td><td>51.71</td><td>4.41 |</td><td>-0.05</td></tr>
<tr><td>107</td><td>A:LEU 107</td><td></td><td>106.14</td><td>21.77 |||</td><td>0.35</td></tr>
<tr><td>108</td><td>A:PHE 108</td><td></td><td>133.22</td><td>98.36 ||||||||</td><td>1.47</td></tr>
<tr><td>110</td><td>A:LYS 110</td><td></td><td>103.23</td><td>0.79 |</td><td>-0.03</td></tr>
<tr><td>117</td><td>A:VAL 117</td><td></td><td>2.26</td><td>0.84 ||||</td><td>0.01</td></tr>
<tr><td>125</td><td>A:LYS 125</td><td></td><td>68.96</td><td>6.05 |</td><td>-0.22</td></tr>
<tr><td>150</td><td>A:TYR 150</td><td>H</td><td>72.17</td><td>29.66 |||||</td><td>-0.03</td></tr>
<tr><td>152</td><td>A:ALA 152</td><td></td><td>45.66</td><td>29.78 |||||||</td><td>0.48</td></tr>
<tr><td>153</td><td>A:ALA 153</td><td></td><td>88.51</td><td>16.86 ||</td><td>0.17</td></tr>
<tr><td>154</td><td>A:ASN 154</td><td></td><td>132.32</td><td>26.45 ||</td><td>-0.14</td></tr>
<tr><td>206</td><td>A:VAL 206</td><td></td><td>69.10</td><td>1.68 |</td><td>0.03</td></tr>
<tr><td>207</td><td>A:GLU 207</td><td></td><td>144.39</td><td>3.19 |</td><td>-0.04</td></tr>
<tr><td>208</td><td>A:HIS 208</td><td>H</td><td>161.44</td><td>139.95 |||||||||</td><td>0.33</td></tr>
<tr><td>209</td><td>A:LEU 209</td><td></td><td>57.01</td><td>26.96 |||||</td><td>0.41</td></tr>
<tr><td>210</td><td>A:PRO 210</td><td></td><td>56.67</td><td>29.58 ||||||</td><td>0.46</td></tr>
<tr><td>225</td><td>A:LEU 225</td><td></td><td>28.92</td><td>3.40 ||</td><td>0.05</td></tr>
<tr><td>241</td><td>A:LEU 241</td><td></td><td>2.51</td><td>0.16 |</td><td>0.00</td></tr>
<tr><td>254</td><td>A:VAL 254</td><td>H</td><td>33.75</td><td>23.59 |||||||</td><td>-0.13</td></tr>
<tr><td>255</td><td>A:TYR 255</td><td></td><td>115.19</td><td>60.49 ||||||</td><td>0.78</td></tr>
<tr><td>256</td><td>A:ASN 256</td><td>H</td><td>59.05</td><td>42.87 ||||||||</td><td>-0.04</td></tr>
<tr><td>257</td><td>A:LEU 257</td><td></td><td>85.71</td><td>42.24 |||||</td><td>0.41</td></tr>
<tr><td>258</td><td>A:GLY 258</td><td></td><td>24.43</td><td>18.72 ||||||||</td><td>0.30</td></tr>
<tr><td>259</td><td>A:ASP 259</td><td></td><td>61.46</td><td>56.66 ||||||||||</td><td>-0.08</td></tr>
<tr><td>260</td><td>A:GLN 260</td><td>H</td><td>34.92</td><td>23.81 |||||||</td><td>-0.27</td></tr>
<tr><td>261</td><td>A:THR 261</td><td></td><td>28.80</td><td>14.22 |||||</td><td>0.23</td></tr>
<tr><td>262</td><td>A:GLY 262</td><td></td><td>41.91</td><td>41.34 ||||||||||</td><td>0.45</td></tr>
<tr><td>263</td><td>A:VAL 263</td><td></td><td>97.35</td><td>47.66 |||||</td><td>0.76</td></tr>
<tr><td>265</td><td>A:LEU 265</td><td></td><td>59.87</td><td>36.93 |||||||</td><td>0.57</td></tr>
<tr><td>266</td><td>A:LYS 266</td><td>H</td><td>170.72</td><td>62.72 ||||</td><td>-0.68</td></tr>
<tr><td>273</td><td>A:VAL 273</td><td></td><td>65.80</td><td>2.35 |</td><td>0.04</td></tr>
<tr><td>316</td><td>A:ARG 316</td><td></td><td>70.96</td><td>53.30 ||||||||</td><td>-0.07</td></tr>
<tr><td>317</td><td>A:ALA 317</td><td></td><td>60.75</td><td>0.37 |</td><td>-0.00</td></tr>
<tr><td>319</td><td>A:THR 319</td><td></td><td>68.29</td><td>53.66 ||||||||</td><td>0.30</td></tr>
<tr><td>320</td><td>A:ASP 320</td><td></td><td>45.32</td><td>18.71 |||||</td><td>-0.09</td></tr>
<tr><td>321</td><td>A:SER 321</td><td></td><td>24.33</td><td>21.86 |||||||||</td><td>0.09</td></tr>
<tr><td>322</td><td>A:GLY 322</td><td></td><td>67.65</td><td>8.19 ||</td><td>0.07</td></tr>
<tr><td>327</td><td>A:VAL 327</td><td></td><td>9.08</td><td>8.40 ||||||||||</td><td>0.13</td></tr>
</table>

b

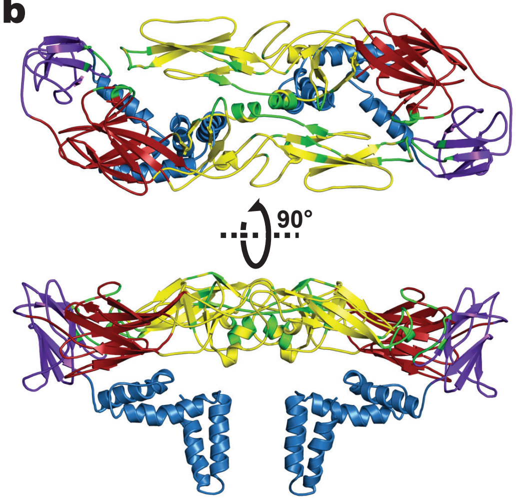

c

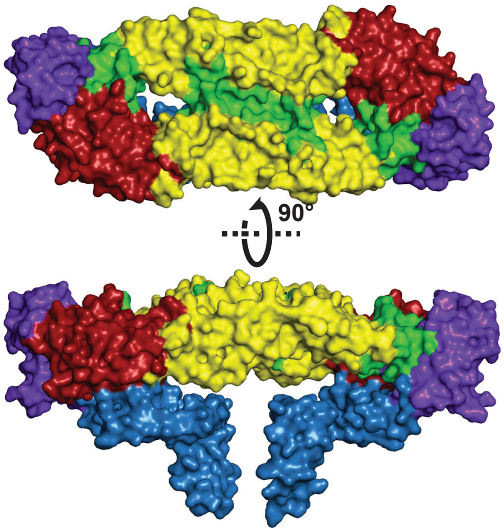

Supplementary Figure 3. E-protein dimer interaction interfaces. (a) Lists of residues forming hydrogen bonds and forming hydrophobic or polar interactions. Residues forming the fusogenic loop (in red frame) from domain II of E-protein are not solvent-accessible. (b) The interfacing residues are shown in green in cartoon representation of the E-protein dimer. E-protein domain I is shown in red, domain II in yellow, domain III in violet, and domain IV in blue. (c) The interfacing residues are shown in green in molecular surface representation of the E-protein dimer.

a

Salt bridges

##	Structure 1	Dist. [Å]	Structure 2
1	M1:LYS 38[NZ]	3.88	E:ASP 222[OD2]

Interface area: 740 Å²

E - M1

Interfacing residues

Inaccessible residues
Solvent-accessible residues
HSDC Residues making Hydrogen/Disulphide bond, Salt bridge or Covalent link
Interfacing residues
ASA Accessible Surface Area, Å² **BSA** Buried Surface Area, Å² **Δ^iG** Solvation energy effect, kcal/mol |||| Buried area percentage, one bar per 10%

##	Structure 1	HSDC	ASA	BSA	Δ^iG
1	M1:VAL 2		168.26	38.57	0.12
3	M1:ILE 4		145.17	13.05	0.21
16	M1:HIS 17		183.77	54.37	0.00
17	M1:LYS 18		117.71	4.17	-0.05
18	M1:TRP 19		220.08	57.75	0.58
19	M1:LEU 20		118.18	58.76	0.94
34	M1:TRP 35		88.91	42.50	0.68
37	M1:LYS 38	S	174.38	39.17	-0.42
38	M1:ASN 39		39.32	9.15	-0.14
40	M1:LEU 41		157.60	38.60	0.62
41	M1:LEU 42		110.91	86.74	1.39
44	M1:ALA 45		43.14	0.33	0.01
45	M1:MET 46		52.12	20.35	0.82
48	M1:VAL 49		39.84	27.85	0.45
51	M1:LEU 52		145.91	7.17	-0.08
52	M1:THR 53		86.91	47.53	0.76
53	M1:LEU 54		167.63	45.81	0.66
62	M1:VAL 63		60.70	17.76	0.28
66	M1:LEU 67		86.54	48.10	0.76
69	M1:LEU 70		126.94	35.68	0.44
70	M1:ALA 71		29.53	12.73	0.20
71	M1:PRO 72		112.27	36.66	0.59

##	Structure 2	HSDC	ASA	BSA	Δ^iG
222	E:ASP 222	S	113.57	35.97	0.18
243	E:GLU 243		87.32	41.83	0.23
244	E:PHE 244		26.70	0.35	-0.00
245	E:GLY 245		20.85	4.02	0.06
246	E:ALA 246		98.50	24.36	-0.15
247	E:PRO 247		23.80	0.77	-0.00
248	E:HIS 248		120.73	16.94	0.27
253	E:ASP 253		65.83	1.84	-0.03
255	E:TYR 255		115.19	36.04	0.48
256	E:ASN 256		59.05	2.94	-0.03
257	E:LEU 257		85.71	27.32	0.44
266	E:LYS 266		170.72	67.16	-0.45
447	E:SER 447		100.39	5.20	-0.06
448	E:ILE 448		85.36	37.20	0.60
449	E:PHE 449		70.73	33.49	0.38
451	E:GLY 451		78.56	16.35	-0.05
452	E:VAL 452		63.01	49.48	0.79
455	E:LEU 455		156.06	25.15	0.40
456	E:PRO 456		46.64	36.08	0.58
459	E:LEU 459		118.40	57.33	0.92
460	E:LEU 460		77.57	42.15	0.67
463	E:ALA 463		46.47	24.33	0.39
464	E:LEU 464		50.05	9.44	0.15
466	E:TRP 466		167.48	16.26	0.26
467	E:LEU 467		85.50	60.04	0.96
470	E:ASN 470		127.69	51.14	-0.48
471	E:MET 471		75.85	14.54	0.45
472	E:ARG 472		205.71	3.19	0.05

b

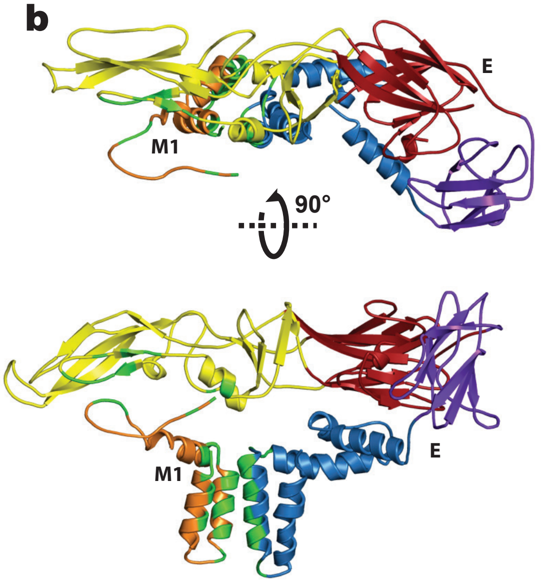

c

Hydrogen bonds

##	Structure 1	Dist. [Å]	Structure 2
1	M2:HIS 7[N]	2.96	E:ALA 267[O]
2	M2:THR 13[OG1]	2.81	E:SER 285[OG]
3	M2:ARG 15[N]	3.87	E:SER 285[OG]
4	M2:ARG 15[NH2]	3.27	E:GLU 411[O]
5	M2:TRP 19[N]	3.44	E:VAL 271[O]
6	M2:HIS 17[O]	2.80	E:VAL 273[N]

Salt bridges

##	Structure 1	Dist. [Å]	Structure 2
1	M2:ARG 15[NE]	3.35	E:GLU 26[OE1]
2	M2:ARG 15[NE]	3.10	E:GLU 26[OE2]
3	M2:ARG 15[NH2]	3.58	E:GLU 26[OE2]

Interface area: 1500 Å²

E - M2

Interfacing residues

Inaccessible residues
Solvent-accessible residues
HSDC Residues making Hydrogen/Disulphide bond, Salt bridge or Covalent link
Interfacing residues
ASA Accessible Surface Area, Å² **BSA** Buried Surface Area, Å² **Δ^iG** Solvation energy effect, kcal/mol |||| Buried area percentage, one bar per 10%

##	Structure 1	HSDC	ASA	BSA	Δ^iG
1	M2:VAL 2		206.91	118.80	1.42
2	M2:LEU 3		156.56	26.53	-0.23
3	M2:ILE 4		151.90	139.01	2.03
4	M2:PRO 5		96.80	43.29	0.43
5	M2:SER 6		68.59	33.81	0.41
6	M2:HIS 7	H	152.70	149.06	1.11
7	M2:ALA 8		65.97	28.85	0.07
8	M2:GLN 9		147.68	64.57	0.15
9	M2:GLY 10		34.91	8.56	0.11
10	M2:GLU 11		135.80	70.63	-0.52
11	M2:LEU 12		157.10	125.24	1.33
12	M2:THR 13	H	124.51	91.15	0.62
13	M2:GLY 14		46.06	25.62	0.06
14	M2:ARG 15	HS	252.67	189.73	-1.41
15	M2:GLY 16		58.65	31.14	0.35
16	M2:HIS 17	H	188.51	57.90	0.05
17	M2:LYS 18		126.40	52.14	0.74
18	M2:TRP 19	H	227.71	161.90	1.60
19	M2:LEU 20		116.07	24.93	0.22
20	M2:GLU 21		105.55	9.05	0.14
23	M2:SER 24		49.20	25.91	0.40
24	M2:LEU 25		124.88	57.02	0.89
27	M2:HIS 28		115.23	40.51	0.37
28	M2:LEU 29		84.45	5.02	0.08
57	M2:VAL 58		137.52	22.60	0.36
58	M2:THR 59		72.76	27.30	0.44
61	M2:ALA 62		57.38	2.01	0.03
65	M2:VAL 66		77.63	10.56	0.17

##	Structure 2	HSDC	ASA	BSA	Δ^iG
8	E:ASN 8		41.58	26.65	-0.10
26	E:GLU 26	S	38.98	24.74	-0.14
27	E:LEU 27		29.91	15.95	-0.18
28	E:GLY 28		51.76	15.67	0.09
61	E:LEU 61		1.85	0.17	0.00
196	E:GLN 196		62.96	5.16	-0.06
197	E:THR 197		9.07	4.39	-0.03
208	E:HIS 208		161.44	0.49	-0.01
209	E:LEU 209		57.01	10.55	0.17
210	E:PRO 210		56.67	16.90	0.27
213	E:TRP 213		13.63	12.85	-0.08
214	E:GLN 214		23.49	19.33	-0.15
215	E:VAL 215		6.58	6.07	0.10
216	E:HIS 216		96.46	50.31	0.55
219	E:TRP 219		82.01	63.26	0.48
222	E:ASP 222		113.57	10.18	-0.12
223	E:LEU 223		35.78	21.25	0.34
224	E:ALA 224		68.55	42.78	0.36
225	E:LEU 225		28.92	25.52	0.41
240	E:ARG 240		93.83	19.25	-0.03
241	E:LEU 241		2.51	2.36	0.04
243	E:GLU 243		87.32	6.08	-0.05
257	E:LEU 257		85.71	16.15	0.26
260	E:GLN 260		34.92	11.11	-0.05
263	E:VAL 263		97.35	29.79	0.48
264	E:LEU 264		11.50	10.83	0.16
265	E:LEU 265		59.87	22.95	0.07
266	E:LYS 266		170.72	8.35	0.05
267	E:ALA 267	H	53.05	50.41	0.33
268	E:LEU 268		15.83	15.83	-0.05
269	E:ALA 269		94.66	76.99	0.54
270	E:GLY 270		84.73	66.40	0.16
271	E:VAL 271	H	39.43	36.75	0.19
272	E:PRO 272		57.98	55.13	0.87
273	E:VAL 273	H	65.80	48.10	0.69
284	E:LYS 284		117.94	35.97	0.08
285	E:SER 285	H	37.95	37.58	-0.07
286	E:GLY 286		8.08	3.18	0.05
287	E:HIS 287		21.90	6.95	-0.63
408	E:LYS 408		43.60	6.78	0.11
411	E:GLU 411	H	113.13	20.20	-0.00
412	E:ARG 412		47.56	9.79	0.15
414	E:THR 414		60.46	19.08	0.05
415	E:VAL 415		104.97	39.72	0.64
416	E:ILE 416		36.94	32.64	0.50
450	E:GLY 450		49.85	2.59	0.04
451	E:GLY 451		78.56	49.15	-0.13
452	E:VAL 452		63.01	2.24	0.04
453	E:GLY 453		39.67	35.15	0.44
454	E:PHE 454		113.79	54.85	0.70
455	E:LEU 455		156.06	76.93	1.23
457	E:LYS 457		32.18	8.19	0.13
458	E:LEU 458		93.99	9.20	0.15
459	E:LEU 459		118.40	10.39	0.17
466	E:TRP 466		167.48	36.10	0.11
469	E:LEU 469		128.24	13.55	0.22

d

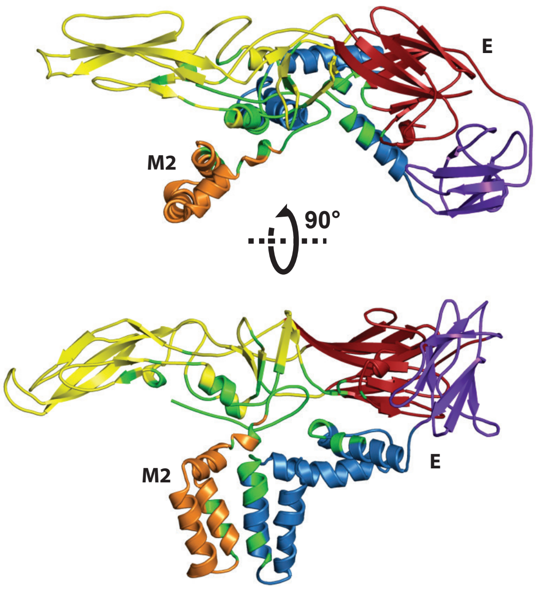

Supplementary Figure 4. E-M interaction interfaces. Interfacing amino acid residues were identified by PDBePISA. Every E-protein interacts with two M-proteins in the heterotetramer. (a, c) The tables show hydrogen bonds, salt bridges, and interfacing amino acids with the buried surface. (b, d) Cartoon representation of E/M-protein interfaces shown in tables. The interfacing residues are shown in green, M-protein in orange, and E-protein domain I in red, domain II in yellow, domain III in violet, and domain IV in blue.

Flavivirus envelope protein alignement

```
Q01299   175| TMGEYGDVSLLCRVASGVDLAQTVILELDKTVEHLPTAWQVHRDWFNDLALPWKHEGA-- | 232 | TBEVH
P14336   175| TMGEYGDVSLLCRVASGVDLAQTVILELDKTVEHLPTAWQVHRDWFNDLALPWKHEGA-- | 232 | TBEVW
P07720   175| TMGDYGDVSLLCRVASGVDLAQTVILELDKTSEHLPTAWQVHRDWFNDLALPWKHEGA-- | 232 | TBEVS
P29837   175| TLGDYGDVSLLCRVASGVDLAQTVVLALDKTHEHLPTAWQVHRDWFNDLALPWKHDGA-- | 232 | LANVT
Q04538   175| RLGDYGDVSLTCKVASGIDVAQTVVMSLDSSKDHLPSAWQVHRDWFEDLALPWKHKDN-- | 232 | POWV
P06935   175| KLGEYGEVTVDCEPRSGIDTSAYYVMSVGE------KSFLVHREWFMDLNLPWSSAGS-- | 226 | WNV
P27395   179| KLGDYGEVTLDCEPRSGLNTEAFYVMTVGS------KSFLVHREWFHDLALPWTSPSS-- | 230 | JEV
P17763   174| QLTDYGALTLDCSPRTGLDFNEMVLLTMEK------KSWLVHKQWFLDLPLPWTSGASTS | 227 | DENV1
P29990   174| ELTGYGTVTMECSPRTGLDFNEMVLLQMEN------KAWLVHRQWFLDLPLPWLPGADTQ | 227 | DENV2
Q6YMS4   172| ILPEYGTLGLECSPRTGLDFNEMILLTMKN------KAWMVHRQWFFDLPLPWASGATTE | 225 | DENV3
Q2YHF0   174| KLPDYGELTLDCEPRSGIDFNEMILMKMKT------KTWLVHKQWFLDLPLPWTAGADTL | 227 | DENV4
P03314   171| EFIGYGKATLECQVQTAVDFGNSYIAEMET------ESWIVDRQWAQDLTLPWQSGSG-- | 222 | YFV
KJ776791 179| TLGGFGSLGLDCEPRTGLDFSDLYYLTMNN------KHWLVHKEWFHDIPLPWHAGADTG | 232 | ZIKV
               :  :*   : *   :.::        :           : *.::*   *:  ***
```

tick-borne flaviviruses (TBEVH, TBEVW, TBEVS, LANVT, POWV)

mosquito-borne flaviviruses (WNV, JEV, DENV1, DENV2, DENV3, DENV4, YFV, ZIKV)

```
Q01299   233| -RNWNNAERLVEFGAPHAVKMDVYNLGDQTGVLLKALAGVPVAHIEGTK---YHLKSGHV | 288 | TBEVH
P14336   233| -QNWNNAERLVEFGAPHAVKMDVYNLGDQTGVLLKALAGVPVAHIEGTK---YHLKSGHV | 288 | TBEVW
P07720   233| -QNWNNAERLVEFGAPHAVKMDVYNLGDQTGVLLKSLAGVPVAHIDGTK---YHLKSGHV | 288 | TBEVS
P29837   233| -EAWNEAGRLVEFGTPHAVKMDVFNLGDQTGVLLKSLAGVPVASIEGTK---YHLKSGHV | 288 | LANVT
Q04538   233| -QDWNSVEKLVEFGPPHAVKMDVFNLGDQTAVLLKSLAGVPLASVEGQK---YHLKSGHV | 288 | POWV
P06935   227| -TTWRNRETLMEFEEPHATKQSVVALGSQEGALHQALAGAIPVEFSSNT---VKLTSGHL | 282 | WNV
P27395   231| -TAWRNRELLMEFEGAHATKQSVVALGSQEGGLHQALAGAIVVEYSS-S---VKLTSGHL | 285 | JEV
P17763   228| QETWNRQDLLVTFKTAHAKKQEVVVLGSQEGAMHTALTGATEIQTSGT----TTIFAGHL | 283 | DENV1
P29990   228| GSNWIQKETLVTFKNPHAKKQDVVVLGSQEGAMHTALTGATEIQMSSG----NLLFTGHL | 283 | DENV2
Q6YMS4   226| TPTWNRKELLVTFKNAHAKKQEVVVLGSQEGAMHTALTGATEIQNSGG----TSIFAGHL | 281 | DENV3
Q2YHF0   228| EVHWNHKERMVTFKVPHAKRQDVTVLGSQEGAMHSALAGATEVDSGDG----NHMFAGHL | 283 | DENV4
P03314   223| -GVWREMHHLVEFEPPHAATIRVLALGNQEGSLKTALTGAMRVTKDTNDNNLYKLHGGHV | 281 | YFV
KJ776791 233| TPHWNNKEALVEFKDAHAKRQTVVVLGSQEGAVHTALAGALEAEMDGAK---GRLSSGHL | 289 | ZIKV
                 *      :: *   **     *   **.* . :  :*:*.              :   **:
```

```
Q01299   401| RVFQKTKKGIERLTVIGEHAWDFGSAGGFLSSIGKALHTVLGGAFNSIFGGVGFLPKLLL | 460 | TBEVH
P14336   401| RVFQKTKKGIERLTVIGEHAWDFGSAGGFLSSIGKAVHTVLGGAFNSIFGGVGFLPKLLL | 460 | TBEVW
P07720   401| RVFQKTRKGIERLTVIGEHAWDFGSTGGFLTSVGKALHTVLGGAFNSLFGGVGFLPKILV | 460 | TBEVS
P29837   401| RVLQKTRKGIERLTVLGEHAWDFGSVGGVMTSIGRAMHTVLGGAFNTLLGGVGFLPKILL | 460 | LANVT
Q04538   402| RMFEKTRRGLERLSVVGEHAWDFGSVGGVLSSVGKAIHTVLGGAFNTLFGGVGFIPKMLL | 461 | POWV
P06935   402| KAFTTTLRGAQRLAALGDTAWDFGSVGGVFTSVGKAIHQVFGGAFRSLFGGMSWITQGLL | 461 | WNV
P27395   405| KAFSTTLKGAQRLAALGDTAWDFGSIGGVFNSIGRAVHQVFGGAFRTLFGGMSWITQGLM | 464 | JEV
P17763   400| KMFEATARGARRMAILGDTAWDFGSIGGVFTSVGKLIHQIFGTAYGVLFSGVSWTMKIGI | 459 | DENV1
P29990   400| QMFETTMRGAKRMAILGDTAWDFGSLGGVFTSIGKALHQVFGAIYGAAFSGVSWTMKILI | 459 | DENV2
Q6YMS4   398| KMFEATERGARRMAILGDTAWDFGSVGGVLNSLGKMVHQIFGSAYTALFSGVSWVMKIGI | 457 | DENV3
Q2YHF0   400| KMFESTYRGAKRMAILGETAWDFGSVGGLLTSLGKAVHQVFGSVYTTMFGGVSWMVRILI | 459 | DENV4
P03314   398| KLFTQTMKGVERLAVMGDTAWDFSSAGGFFTSVGKGIHTVFGSAFQGLFGGLNWITKVIM | 457 | YFV
KJ776791 409| KAFEATVRGAKRMAVLGDTAWDFGSVGGALNSLGKGIHQIFGAAFKSLFGGMSWFSQILI | 468 | ZIKV
              : :   * :* .*:: :*: ****.* ** :.*:*: :* ::*  :    :.*:.:  :  :
```

Flavivirus membrane protein alignement

```
Q01299   1 | SVLIPSHAQGELTGRGHKWLEGDSLRTHLTRVEGWVWKNRLLALAMVTVVWLTLESVVTR | 60 | TBEVH
P14336   1 | SVLIPSHAQGELTGRGHKWLEGDSLRTHLTRVEGWVWKNKLLALAMVTVVWLTLESVVTR | 60 | TBEVW
P07720   1 | SVLIPSHAQGDLTGRGHKWLEGDSLRTHLTRVEGWVWKNKVLTLAVIAVVWLTVESVVTR | 60 | TBEVS
P29837   1 | SVLIPSHAQRDLTGRGHQWLEGEAVKAHLTRVEGWVWKNKLFTLSLVMVAWLMVDGLLPR | 60 | LANVT
Q04538   1 | SVVIPTHAQKDMVGRGHAWLKGDNIRDHVTRVEGWMWKNKLLTAAIVALAWLMVDSWMAR | 60 | POWV
P06935   1 | SLTVQTHGESTLANKKGAWLDSTKATRYLVKTESWILRNPGYALVAAVIGWMLGSNTMQR | 60 | WNV
P27395   1 | SVSVQTHGESSLVNKKEAWLDSTKATRYLMKTENWIIRNPGYAFLAAVLGWMLGSNNGQR | 60 | JEV
P17763   1 | SVALAPHVGLGLETRTETWMSSEGAWKQIQKVETWALRHPGFTVIALFLAHAIGTSITQK | 60 | DENV1
P29990   1 | SVALVPHVGMGLETRTETWMSSEGAWKHVQRIETWILRHPGFTMMAAILAYTIGTTHFQR | 60 | DENV2
Q6YMS4   1 | SVALAPHVGMGLDTRTQTWMSAEGAWRQVEKVETWALRHPGFTILALFLAHYIGTSLTQK | 60 | DENV3
Q2YHF0   1 | SVALTPHSGMGLETRAETWMSSEGAWKHAQRVESWILRNPGFALLAGFMAYMIGQTGIQR | 60 | DENV4
P03314   1 | AIDLPTHENHGLKTRQEKWMTGRMGERQLQKIERWFVRNPFFAVTALTIAYLVGSNMTQR | 60 | YFV
KJ776791 1 | AVTLPSHSTRKLQTRSQTWLESREYTKHLIRVENWIFRNPGFALAAAAIAWLLGSSTSQK | 60 | ZIKV
             :: :   *     :   :    *:  .          : * *  ::    :      :           :
```

Supplementary Figure 5. Multiple sequence alignment of flavivirus envelope and membrane proteins. Sequences were aligned using Clustal Omega. Parts of the alignments are shown in the figure. Histidines that may be involved in the putative pH-dependent conformational switch are highlighted in red. Uniprot IDs are provided on the left side of the sequences (except for ZIKV, where Genbank ID is shown). The position of the amino acids in the aligned sequence and the virus names are shown on the right. TBEVH – Tick-borne encephalitis virus strain Hypr; TBEVW - Tick-borne encephalitis virus European subtype (strain Neudoerfl); TBEVS - Tick-borne encephalitis virus Far Eastern subtype (strain Sofjin); LANVT - Langat virus (strain TP21); POWV - Tick-borne powassan virus (strain LB); WNV - West Nile virus (strain 956); JEV - Japanese encephalitis virus (strain SA-14); DENV1 - Dengue virus type 1 (strain Nauru/West Pac/1974); DENV2 - Dengue virus type 2 (strain Thailand-/16681/1984); DENV3 - Dengue virus type 3 (strain Sri Lanka/1266/2000); DENV4 - Dengue virus type 4 (strain Thailand-/0348/1991); YFV - Yellow fever virus (strain 17D vaccine); ZIKV - Zika virus (strain H/PF/2013). Conservation of a residue is denoted by: "*" - absolute conservation; ":" - conservation of amino acids with strongly similar properties; "." – conservation of amino acids with weakly similar properties.

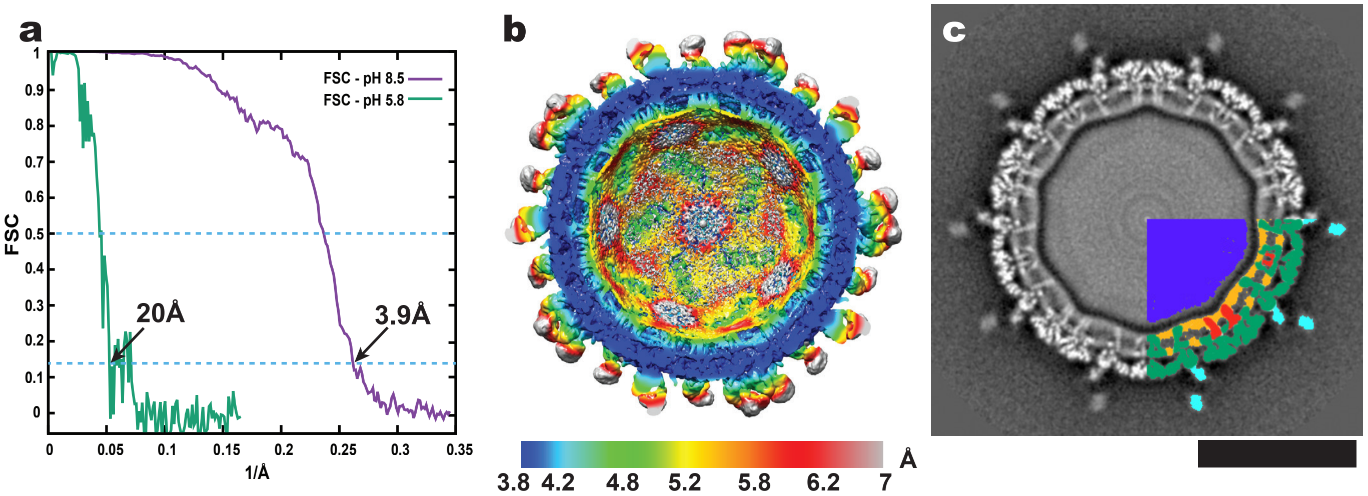

Supplementary Figure 6. Cryo-EM reconstruction of TBEV Fab 19/1786 complex. (a) Fourier shell correlation (FSC) curves of final reconstructions of TBEV-Fab 19/1786 complex at pH 8.5 (purple) and at pH 5.8 (green) calculated according to “gold standard”. (b) Local resolution of cryo-EM map of TBEV-Fab 19/1786 complex. The display shows a cut-away half map colored according to the local resolution. Parts of the map with resolution worse than 7 Å are shown in grey. The non-sharpened electron density map was used for the display. (c) Central slice of electron-density map perpendicular to virus fivefold axis. The overall shape and features of the TBEV particle remained intact after attachment of Fab 19/1786 fragment. The lower right quadrant of the slice is color-coded as follows: nucleocapsid – blue; inner and outer membrane leaflets – orange; M-proteins – red; E-proteins – green; Fab 19/1786 attached to virus surface – cyan.

a

E-protein domain III - Fab 1786 heavy chain interfaces

Hydrogen bonds

##	Fab 19/1786 H	Dist. [Å]	Domain III
1	H:ASN 52[ND2]	3.61	B:MET 306[O]
2	H:ASN 52[ND2]	3.01	B:CYS 307[O]
3	H:TYR 101[OH]	3.89	B:GLU 387[OE1]
4	H:TYR 101[OH]	3.74	B:GLU 387[OE2]
5	H:TYR 101[O]	3.45	B:THR 310[OG1]
6	H:SER 57[OG]	3.59	B:ARG 339[NH2]

Salt bridges

##	Fab 19/1786 H	Dist. [Å]	Domain III
1	H:ASP 104[OD1]	3.75	B:LYS 311[NZ]

Interface area: 445 Å^2

Interfacing residues

Inaccessible residues

Solvent-accessible residues

HSDC Residues making Hydrogen/Disulphide bond, Salt bridge or Covalent link

Interfacing residues

ASA Accessible Surface Area, Å² **BSA** Buried Surface Area, Å² $\Delta^{i}G$ Solvation energy effect, kcal/mol |||| Buried area percentage, one bar per 10%

<table>
<tr><th>##</th><th>Fab 19/1786 H</th><th>HSDC</th><th>ASA</th><th>BSA</th><th>ΔiG</th></tr>
<tr><td>1</td><td>H:SER 31</td><td></td><td>54.91</td><td>0.85 |</td><td>-0.01</td></tr>
<tr><td>2</td><td>H:THR 51</td><td></td><td>8.39</td><td>0.34 |</td><td>0.01</td></tr>
<tr><td>3</td><td>H:ASN 52</td><td>H</td><td>54.84</td><td>48.93 |||||||||</td><td>-0.08</td></tr>
<tr><td>4</td><td>H:SER 53</td><td></td><td>40.81</td><td>24.11 ||||||</td><td>-0.28</td></tr>
<tr><td>5</td><td>H:ASP 54</td><td></td><td>97.66</td><td>11.05 ||</td><td>-0.01</td></tr>
<tr><td>6</td><td>H:ASP 56</td><td></td><td>98.04</td><td>30.36 ||||</td><td>-0.02</td></tr>
<tr><td>7</td><td>H:SER 57</td><td>H</td><td>75.49</td><td>51.06 |||||||</td><td>0.32</td></tr>
<tr><td>8</td><td>H:THR 58</td><td></td><td>73.76</td><td>8.60 ||</td><td>-0.03</td></tr>
<tr><td>9</td><td>H:TYR 59</td><td></td><td>124.73</td><td>38.32 ||||</td><td>0.59</td></tr>
<tr><td>10</td><td>H:LYS 65</td><td></td><td>134.94</td><td>26.65 ||</td><td>-0.37</td></tr>
<tr><td>11</td><td>H:TYR 101</td><td>H</td><td>166.52</td><td>41.56 |||</td><td>0.06</td></tr>
<tr><td>12</td><td>H:ASP 102</td><td></td><td>106.40</td><td>43.33 |||||</td><td>-0.05</td></tr>
<tr><td>13</td><td>H:TYR 103</td><td></td><td>93.11</td><td>69.01 ||||||||</td><td>0.73</td></tr>
<tr><td>14</td><td>H:ASP 104</td><td>S</td><td>77.12</td><td>20.33 |||</td><td>-0.10</td></tr>
<tr><td>15</td><td>H:GLY 105</td><td></td><td>48.45</td><td>13.35 |||</td><td>0.20</td></tr>
</table>

<table>
<tr><th>##</th><th>Domain III</th><th>HSDC</th><th>ASA</th><th>BSA</th><th>ΔiG</th></tr>
<tr><td>1</td><td>B:THR 303</td><td></td><td>131.99</td><td>33.27 |||</td><td>0.19</td></tr>
<tr><td>2</td><td>B:TYR 304</td><td></td><td>71.20</td><td>6.25 |</td><td>0.10</td></tr>
<tr><td>3</td><td>B:THR 305</td><td></td><td>92.83</td><td>68.16 ||||||||</td><td>1.00</td></tr>
<tr><td>4</td><td>B:MET 306</td><td>H</td><td>108.81</td><td>49.59 |||||</td><td>0.51</td></tr>
<tr><td>5</td><td>B:CYS 307</td><td>H</td><td>16.98</td><td>15.25 |||||||||</td><td>-0.12</td></tr>
<tr><td>6</td><td>B:ASP 308</td><td></td><td>49.25</td><td>45.23 ||||||||||</td><td>0.11</td></tr>
<tr><td>7</td><td>B:LYS 309</td><td></td><td>109.03</td><td>63.74 ||||||</td><td>0.91</td></tr>
<tr><td>8</td><td>B:THR 310</td><td>H</td><td>84.83</td><td>48.08 ||||||</td><td>0.37</td></tr>
<tr><td>9</td><td>B:LYS 311</td><td>S</td><td>96.52</td><td>46.86 |||||</td><td>0.44</td></tr>
<tr><td>10</td><td>B:THR 335</td><td></td><td>103.30</td><td>41.76 |||||</td><td>0.58</td></tr>
<tr><td>11</td><td>B:ARG 339</td><td>H</td><td>84.66</td><td>13.17 ||</td><td>-0.15</td></tr>
<tr><td>12</td><td>B:GLU 387</td><td>H</td><td>125.79</td><td>30.68 |||</td><td>-0.35</td></tr>
</table>

E-protein domain III - Fab 1786 light chain interfaces

Hydrogen bonds

##	Fab 19/1786 L	Dist. [Å]	Domain III
1	L:ASN 32[ND2]	2.92	B:SER 333[O]
2	L:ASN 32[ND2]	3.75	B:GLY 334[O]
3	L:HIS 94[N]	3.25	B:LYS 336[O]
4	L:HIS 94[ND1]	3.48	B:PRO 337[O]
5	L:ASN 92[O]	2.69	B:LYS 336[N]
6	L:ASN 32[OD1]	3.68	B:LYS 336[NZ]

Interface area: 300 Å^2

Interfacing residues

Inaccessible residues

Solvent-accessible residues

HSDC Residues making Hydrogen/Disulphide bond, Salt bridge or Covalent link

Interfacing residues

ASA Accessible Surface Area, Å² **BSA** Buried Surface Area, Å² $\Delta^{i}G$ Solvation energy effect, kcal/mol |||| Buried area percentage, one bar per 10%

<table>
<tr><th>##</th><th>Fab 19/1786 L</th><th>HSDC</th><th>ASA</th><th>BSA</th><th>ΔiG</th></tr>
<tr><td>1</td><td>L:ILE 2</td><td></td><td>5.34</td><td>1.01 ||</td><td>0.02</td></tr>
<tr><td>2</td><td>L:GLN 27</td><td></td><td>107.63</td><td>22.99 |||</td><td>-0.26</td></tr>
<tr><td>3</td><td>L:ASN 28</td><td></td><td>120.25</td><td>0.25 |</td><td>-0.00</td></tr>
<tr><td>4</td><td>L:THR 31</td><td></td><td>81.87</td><td>13.01 ||</td><td>-0.15</td></tr>
<tr><td>5</td><td>L:ASN 32</td><td>H</td><td>34.04</td><td>29.66 |||||||||</td><td>-0.37</td></tr>
<tr><td>6</td><td>L:TYR 49</td><td></td><td>112.43</td><td>3.26 |</td><td>0.05</td></tr>
<tr><td>7</td><td>L:SER 50</td><td></td><td>28.13</td><td>5.76 |||</td><td>-0.07</td></tr>
<tr><td>8</td><td>L:TYR 91</td><td></td><td>80.21</td><td>47.86 ||||||</td><td>0.27</td></tr>
<tr><td>9</td><td>L:ASN 92</td><td>H</td><td>69.40</td><td>61.69 |||||||||</td><td>-0.66</td></tr>
<tr><td>10</td><td>L:ASN 93</td><td></td><td>43.50</td><td>22.49 ||||||</td><td>-0.09</td></tr>
<tr><td>11</td><td>L:HIS 94</td><td>H</td><td>163.14</td><td>91.29 ||||||</td><td>0.05</td></tr>
<tr><td>12</td><td>L:LEU 96</td><td></td><td>79.29</td><td>13.82 ||</td><td>0.22</td></tr>
</table>

<table>
<tr><th>##</th><th>Domain III</th><th>HSDC</th><th>ASA</th><th>BSA</th><th>ΔiG</th></tr>
<tr><td>1</td><td>B:TYR 304</td><td></td><td>71.20</td><td>4.36 |</td><td>0.07</td></tr>
<tr><td>2</td><td>B:THR 305</td><td></td><td>92.83</td><td>15.67 ||</td><td>-0.13</td></tr>
<tr><td>3</td><td>B:LYS 311</td><td></td><td>96.52</td><td>30.16 ||||</td><td>-0.42</td></tr>
<tr><td>4</td><td>B:SER 333</td><td>H</td><td>77.16</td><td>30.67 ||||</td><td>-0.04</td></tr>
<tr><td>5</td><td>B:GLY 334</td><td>H</td><td>21.89</td><td>21.89 ||||||||||</td><td>0.04</td></tr>
<tr><td>6</td><td>B:THR 335</td><td></td><td>103.30</td><td>61.54 ||||||</td><td>0.91</td></tr>
<tr><td>7</td><td>B:LYS 336</td><td>H</td><td>73.52</td><td>52.74 ||||||||</td><td>-0.47</td></tr>
<tr><td>8</td><td>B:PRO 337</td><td>H</td><td>29.55</td><td>20.44 |||||||</td><td>0.06</td></tr>
<tr><td>9</td><td>B:CYS 338</td><td></td><td>2.44</td><td>2.26 ||||||||||</td><td>0.04</td></tr>
<tr><td>10</td><td>B:ASN 366</td><td></td><td>119.82</td><td>53.72 |||||</td><td>-0.11</td></tr>
</table>

E-protein domain I - Fab 1786 heavy chain interfaces

Hydrogen bonds

##	Domain I	Dist. [Å]	Fab 19/1786 H
1	B:LYS 161[NZ]	3.86	H:GLY 66[O]
2	B:GLU 51[OE1]	2.97	H:ARG 74[NE]
3	B:THR 156[OG1]	3.77	H:GLN 82[NE2]

Salt bridges

##	Domain I	Dist. [Å]	Fab 19/1786 H
1	B:GLU 51[OE1]	2.97	H:ARG 74[NE]

Interface area: 300 Å^2

Interfacing residues

Inaccessible residues

Solvent-accessible residues

HSDC Residues making Hydrogen/Disulphide bond, Salt bridge or Covalent link

Interfacing residues

ASA Accessible Surface Area, Å² **BSA** Buried Surface Area, Å² $\Delta^{i}G$ Solvation energy effect, kcal/mol |||| Buried area percentage, one bar per 10%

<table>
<tr><th>##</th><th>Domain I</th><th>HSDC</th><th>ASA</th><th>BSA</th><th>ΔiG</th></tr>
<tr><td>1</td><td>B:GLU 51</td><td>HS</td><td>93.32</td><td>58.79 |||||||</td><td>-0.49</td></tr>
<tr><td>2</td><td>B:ASN 52</td><td></td><td>80.76</td><td>14.14 ||</td><td>-0.06</td></tr>
<tr><td>3</td><td>B:LYS 136</td><td></td><td>113.50</td><td>0.70 |</td><td>-0.02</td></tr>
<tr><td>4</td><td>B:THR 156</td><td>H</td><td>114.48</td><td>26.21 |||</td><td>-0.06</td></tr>
<tr><td>5</td><td>B:SER 158</td><td></td><td>115.29</td><td>43.90 ||||</td><td>0.32</td></tr>
<tr><td>6</td><td>B:ARG 160</td><td></td><td>49.39</td><td>4.42 |</td><td>-0.05</td></tr>
<tr><td>7</td><td>B:LYS 161</td><td>H</td><td>76.90</td><td>37.79 |||||</td><td>-1.09</td></tr>
<tr><td>8</td><td>B:THR 162</td><td></td><td>60.72</td><td>1.29 |</td><td>0.00</td></tr>
<tr><td>9</td><td>B:GLY 177</td><td></td><td>47.56</td><td>6.69 ||</td><td>0.11</td></tr>
<tr><td>10</td><td>B:GLU 277</td><td></td><td>110.47</td><td>18.69 ||</td><td>-0.12</td></tr>
<tr><td>11</td><td>B:GLY 278</td><td></td><td>45.53</td><td>3.35 |</td><td>0.05</td></tr>
<tr><td>12</td><td>B:THR 279</td><td></td><td>72.04</td><td>60.83 |||||||||</td><td>0.80</td></tr>
<tr><td>13</td><td>B:LYS 280</td><td></td><td>60.86</td><td>11.88 ||</td><td>0.19</td></tr>
<tr><td>14</td><td>B:ASN 367</td><td></td><td>150.92</td><td>20.75 ||</td><td>-0.24</td></tr>
</table>

<table>
<tr><th>##</th><th>Fab 19/1786 H</th><th>HSDC</th><th>ASA</th><th>BSA</th><th>ΔiG</th></tr>
<tr><td>1</td><td>H:GLY 15</td><td></td><td>56.50</td><td>7.44 ||</td><td>0.03</td></tr>
<tr><td>2</td><td>H:GLY 66</td><td>H</td><td>73.45</td><td>24.48 ||||</td><td>-0.04</td></tr>
<tr><td>3</td><td>H:ARG 67</td><td></td><td>61.35</td><td>7.50 ||</td><td>-0.07</td></tr>
<tr><td>4</td><td>H:PHE 68</td><td></td><td>10.03</td><td>4.79 |||||</td><td>-0.05</td></tr>
<tr><td>5</td><td>H:THR 69</td><td></td><td>74.43</td><td>22.77 ||||</td><td>-0.13</td></tr>
<tr><td>6</td><td>H:ASP 73</td><td></td><td>48.92</td><td>28.39 ||||||</td><td>-0.27</td></tr>
<tr><td>7</td><td>H:ARG 74</td><td>HS</td><td>147.42</td><td>64.68 |||||</td><td>-0.53</td></tr>
<tr><td>8</td><td>H:ALA 75</td><td></td><td>82.66</td><td>37.18 |||||</td><td>0.57</td></tr>
<tr><td>9</td><td>H:LYS 76</td><td></td><td>149.25</td><td>30.64 |||</td><td>0.46</td></tr>
<tr><td>10</td><td>H:GLN 82</td><td>H</td><td>76.49</td><td>25.05 ||||</td><td>-0.08</td></tr>
<tr><td>11</td><td>H:SER 84</td><td></td><td>43.78</td><td>29.69 |||||||</td><td>0.20</td></tr>
<tr><td>12</td><td>H:SER 85</td><td></td><td>59.67</td><td>12.31 |||</td><td>0.07</td></tr>
</table>

b

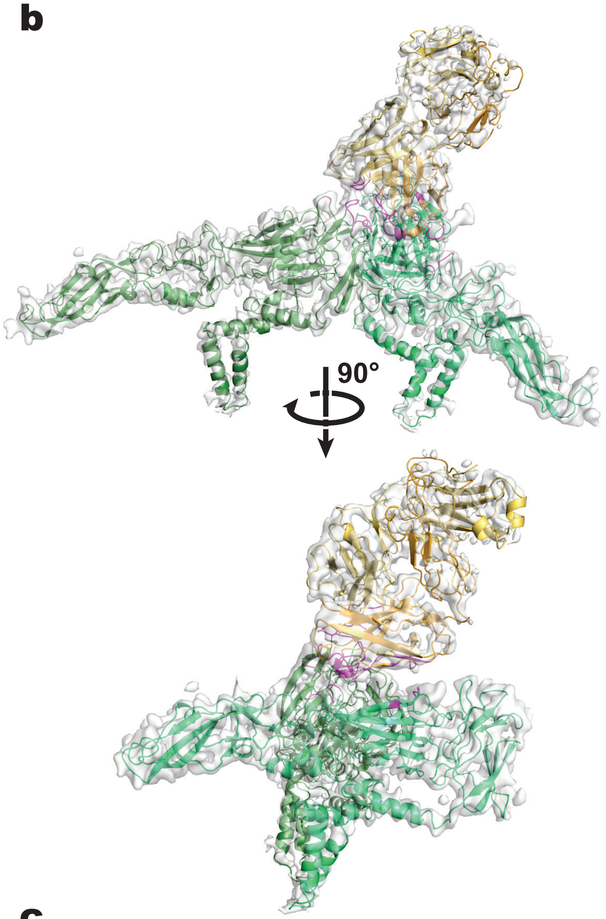

c

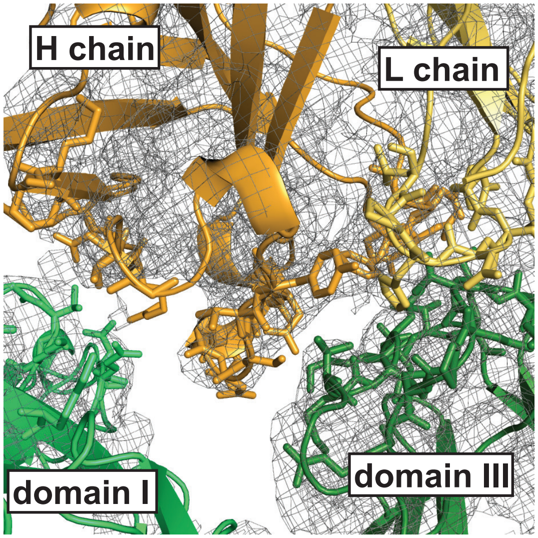

Supplementary Figure 7. E-protein – Fab 19/1786 interaction interfaces next to threefold axis. Interfacing amino acids were identified using PDBePISA. (a) Tables show hydrogen bonds, salt bridges, and amino acids with buried surface. (b) Cartoon representation of E-protein – Fab 19/1786 interfaces shown in tables. E-proteins are shown in green, Fab 19/1786 heavy chain in orange, Fab 19/1786 light chain in yellow, and residues forming the interface in magenta. (c) Detail of interaction interface.

a

E-protein domain III - Fab 1786 heavy chain interfaces

Hydrogen bonds

##	Fab 19/1786 H	Dist. [Å]	Domain III
1	H:SER 53[OG]	3.58	C:LYS 309[NZ]
2	H:ASP 56[OD2]	2.82	C:LYS 309[NZ]
3	H:SER 53[O]	2.90	C:LYS 309[NZ]
4	H:TYR 101[O]	3.87	C:THR 310[OG1]
5	H:SER 57[OG]	3.84	C:ARG 339[NH2]

Salt bridges

##	Fab 19/1786 H	Dist. [Å]	Domain III
1	H:ASP 56[OD2]	2.82	C:LYS 309[NZ]
2	H:ASP 104[OD1]	3.90	C:LYS 311[NZ]

Interface area: 440 Å^2

Interfacing residues

Inaccessible residues
Solvent-accessible residues
HSDC Residues making Hydrogen/Disulphide bond, Salt bridge or Covalent link
Interfacing residues
ASA Accessible Surface Area, Å² **BSA** Buried Surface Area, Å² **$\Delta^{i}G$** Solvation energy effect, kcal/mol |||| Buried area percentage, one bar per 10%

##	Fab 19/1786 H	HSDC	ASA	BSA	$\Delta^{i}G$
1	H:SER 30		57.11	5.75	-0.07
2	H:SER 31		52.54	9.86	0.12
3	H:ASN 52		53.02	45.59	0.04
4	H:SER 53	H	48.62	34.92	-0.34
5	H:ASP 54		66.83	3.82	0.06
6	H:ASP 56	HS	117.62	22.50	-0.10
7	H:SER 57	H	73.05	51.64	0.27
8	H:THR 58		77.33	11.91	-0.14
9	H:TYR 59		108.26	26.33	0.42
10	H:LYS 65		125.17	12.99	-0.41
11	H:TYR 101	H	167.57	35.60	0.10
12	H:ASP 102		101.93	43.78	-0.01
13	H:TYR 103		102.14	78.44	0.84
14	H:ASP 104	S	85.25	25.17	-0.14
15	H:GLY 105		49.72	15.63	0.24

##	Domain III	HSDC	ASA	BSA	$\Delta^{i}G$
1	C:THR 303		128.43	12.34	0.17
2	C:TYR 304		75.44	2.02	-0.00
3	C:THR 305		87.47	58.32	0.93
4	C:MET 306		95.61	48.76	0.44
5	C:CYS 307		16.54	16.18	-0.11
6	C:ASP 308		50.24	44.08	0.14
7	C:LYS 309	HS	107.40	76.31	-1.23
8	C:THR 310	H	85.27	45.35	0.40
9	C:LYS 311	S	100.62	44.28	0.31
10	C:PHE 332		8.09	0.31	0.00
11	C:GLY 334		28.90	9.08	0.15
12	C:THR 335		105.67	49.87	0.55
13	C:ARG 339	H	79.45	10.42	-0.12
14	C:GLU 387		129.87	34.18	-0.44

E-protein domain III - Fab 1786 light chain interfaces

Hydrogen bonds

##	Fab 19/1786 L	Dist. [Å]	Domain III
1	L:ASN 32[ND2]	2.81	C:SER 333[O]
2	L:ASN 32[ND2]	3.56	C:GLY 334[O]
3	L:HIS 94[N]	3.53	C:LYS 336[O]
4	L:HIS 94[ND1]	3.09	C:LYS 336[O]
5	L:HIS 94[ND1]	3.76	C:PRO 337[O]
6	L:TYR 91[O]	3.85	C:LYS 336[N]
7	L:ASN 32[OD1]	3.54	C:LYS 336[NZ]
8	L:ASN 92[O]	3.68	C:ASN 366[ND2]

Interface area: 275 Å^2

Interfacing residues

Inaccessible residues
Solvent-accessible residues
HSDC Residues making Hydrogen/Disulphide bond, Salt bridge or Covalent link
Interfacing residues
ASA Accessible Surface Area, Å² **BSA** Buried Surface Area, Å² **$\Delta^{i}G$** Solvation energy effect, kcal/mol |||| Buried area percentage, one bar per 10%

##	Fab 19/1786 L	HSDC	ASA	BSA	$\Delta^{i}G$
1	L:ILE 2		5.45	0.65	0.01
2	L:GLN 27		103.52	10.03	-0.11
3	L:THR 31		87.81	5.16	-0.06
4	L:ASN 32	H	41.31	35.50	-0.44
5	L:SER 50		33.75	0.86	-0.01
6	L:TYR 91	H	75.48	44.15	0.24
7	L:ASN 92	H	78.83	64.26	-0.57
8	L:ASN 93		52.04	24.09	-0.08
9	L:HIS 94	H	157.42	91.55	0.11
10	L:LEU 96		89.25	14.87	0.24

##	Domain III	HSDC	ASA	BSA	$\Delta^{i}G$
1	C:THR 305		87.47	24.87	0.28
2	C:LYS 311		100.62	1.66	-0.01
3	C:SER 333	H	73.07	24.86	-0.12
4	C:GLY 334	H	28.90	19.82	0.06
5	C:THR 335		105.67	55.80	0.83
6	C:LYS 336	H	88.19	64.80	-0.20
7	C:PRO 337	H	38.77	28.00	0.24
8	C:CYS 338		0.16	0.16	0.00
9	C:ASN 366	H	115.96	40.10	-0.22

E-protein domain II - Fab 1786 heavy chain interfaces

Hydrogen bonds

##	Domain II	Dist. [Å]	Fab 19/1786 H
1	B:LYS 69[NZ]	2.09	H:GLY 55[O]
2	B:LYS 69[NZ]	3.04	H:ASP 54[O]
3	B:GLU 84[N]	3.30	H:ASP 56[OD1]
4	B:VAL 70[O]	3.40	H:ARG 74[NH2]

Interface area: 365 Å^2

Interfacing residues

Inaccessible residues
Solvent-accessible residues
HSDC Residues making Hydrogen/Disulphide bond, Salt bridge or Covalent link
Interfacing residues
ASA Accessible Surface Area, Å² **BSA** Buried Surface Area, Å² **$\Delta^{i}G$** Solvation energy effect, kcal/mol |||| Buried area percentage, one bar per 10%

##	Domain II	HSDC	ASA	BSA	$\Delta^{i}G$
1	B:LYS 64		97.54	1.97	-0.07
2	B:SER 66		44.45	10.21	0.16
3	B:ASP 67		80.90	64.57	-0.64
4	B:LYS 69	H	102.89	77.40	-0.71
5	B:VAL 70	H	43.91	9.44	-0.08
6	B:ALA 71		15.69	14.23	0.23
7	B:ALA 72		25.67	4.02	-0.05
8	B:ARG 73		102.46	6.97	-0.15
9	B:ALA 80		8.03	0.67	0.01
10	B:THR 81		125.01	1.35	-0.02
11	B:LEU 82		29.64	21.59	0.35
12	B:ALA 83		79.73	41.44	0.55
13	B:GLU 84	H	29.92	28.13	-0.09
14	B:GLN 87		96.17	39.08	-0.40
15	B:LYS 118		68.84	22.04	0.32
16	B:LYS 251		126.74	15.88	-0.07

##	Fab 19/1786 H	HSDC	ASA	BSA	$\Delta^{i}G$
1	H:SER 53		48.62	0.12	-0.00
2	H:ASP 54	H	66.83	41.46	-0.38
3	H:GLY 55	H	35.40	27.59	-0.28
4	H:ASP 56	H	117.62	103.65	-0.47
5	H:SER 57		73.05	16.53	0.25
6	H:THR 58		77.33	20.00	0.30
7	H:GLY 66		65.11	1.84	-0.02
8	H:THR 69		80.04	14.57	0.23
9	H:ILE 70		10.93	7.37	-0.08
10	H:SER 71		57.12	21.43	0.34
11	H:ARG 72		35.51	24.51	-0.06
12	H:ASP 73		38.34	0.37	-0.00
13	H:ARG 74	H	158.46	74.33	-1.19
14	H:ALA 75		87.91	8.02	0.13
15	H:LYS 76		141.42	7.69	0.12

b

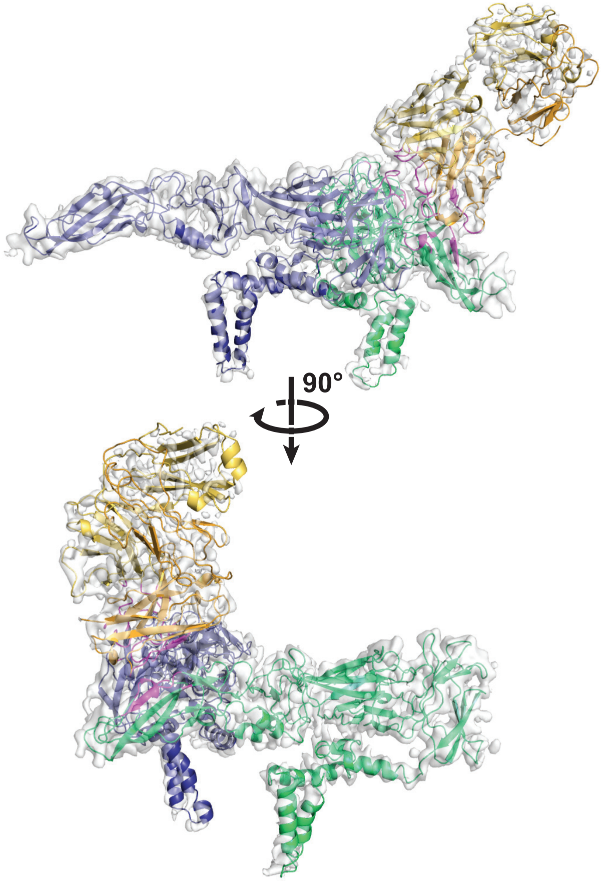

c

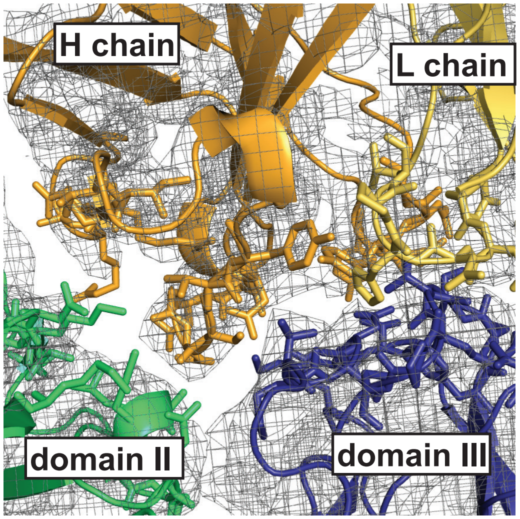

Supplementary Figure 8. E-protein – Fab 19/1786 interaction interfaces close to fivefold axis. Interfacing amino acid residues were identified using PDBePISA. (a) Tables show hydrogen bonds, salt bridges, and amino acids with buried surface. (b) Cartoon representation of E-protein – Fab 19/1786 interfaces show in tables. E-proteins are shown in green and blue, Fab 19/1786 heavy chain in orange, Fab 19/1786 light chain in yellow, and residues forming the interface in magenta. (c) Detail of interaction interface.

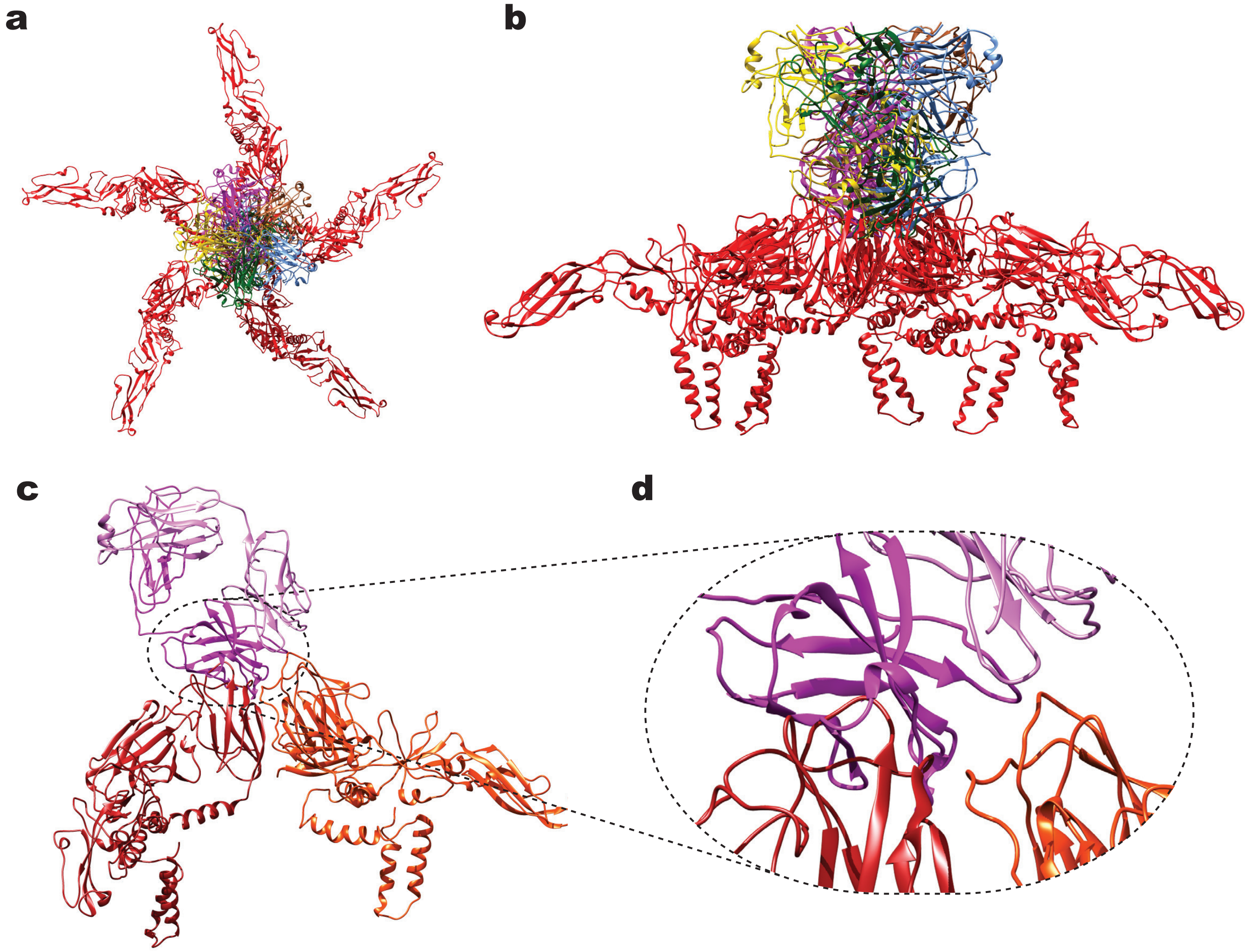

Supplementary Figure 9. Steric hindrance prevents attachment of Fab 19/1786 fragments to unoccupied E-proteins. Molecular model shows clashes of Fab fragments with each other caused by simulated attachment of Fab 19/1786 to unoccupied E-proteins close to fivefold axis (a, b). The Fab 19/1786 could not bind to the third E-protein within the icosahedral asymmetric unit because upon binding to domain III of the unoccupied E-protein, the heavy chain of the Fab would clash with domain III of a neighboring E-protein (c) and (d). E-proteins are shown in red; Fab 19/1786 fragments in multiple colors.

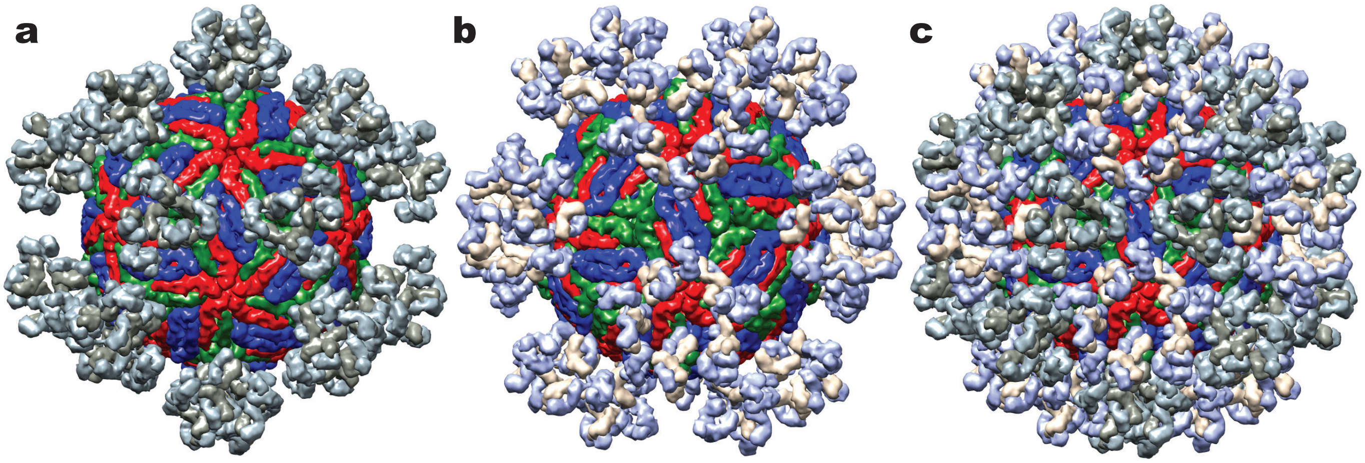

Supplementary Figure 10. TBEV particle fully occupied by mouse IgG1 antibodies. Model of TBEV virion covered with IgG1 antibodies on sites corresponding to epitopes of Fab 19/1786. PDB:1IGY was superposed onto Fab 19/1786 attached to virion surface next to virus threefold axis (a), close to virus fivefold axis (b), and on both interaction sites (c). The attachment produces clash-free coverage of the particle with IgG1.
